# Therapeutic Potential of Extracellular Vesicles: From Biogenesis, Isolation and Molecular Characterization to Addressing Translational Gaps and Regulatory Barriers

**DOI:** 10.3390/ijms27041676

**Published:** 2026-02-09

**Authors:** Dragan Primorac, Petar Brlek, Luka Bulić, Nenad Hrvatin, Vedrana Škaro, Petar Projić, Martina Glavan, Ijeoma Oleru, Pierre Rocheteau, Carlo Tremolada, Ariana DeMers, Mary A. Ambach, Don Buford, Tamara Knežević, Dimitrios Kouroupis, Cole Conforti, D. Wood Kimbrough, R. Peter Schnorr, Lindsay Williams, Raminta Vaiciuleviciute, Žan Fortuna, Lara Oprešnik, Blaž Curk, Miomir Knežević, Gordana Kalan Živčec, Adelina Hrkać, Dimitrios Tsoukas, Ilona Uzieliene, Jolita Pachaleva, Eiva Bernotiene, Kristiana Barbato, Neep Patel, Isabella Demirdjian Guanche, Evangelos V. Badiavas, Jana Mešić, Ana Medić Flajšman, Romina Milanič, Danijela Klarić, Vasiliki E. Kalodimou, Massimo Allegri, Johannes Brachmann, Wei Seong Toh, Nancy Duarte Delgado, Ali Mobasheri

**Affiliations:** 1 St. Catherine Specialty Hospital, 10000 Zagreb, Croatia; dragan.primorac@svkatarina.hr (D.P.); luka.bulic@svkatarina.hr (L.B.); nenad.hrvatin@svkatarina.hr (N.H.); tamara.knezevic@svkatarina.hr (T.K.); adelina.surjan@gmail.com (A.H.); jana.mesic@svkatarina.hr (J.M.); ana.medicflajsman@svkatarina.hr (A.M.F.); romina.milanic@svkatarina.hr (R.M.); danijela.klaric@svkatarina.hr (D.K.); 2 International Center for Applied Biological Research, 10000 Zagreb, Croatia; vedrana.skaro@icabs.eu (V.Š.); petar.projic@icabs.eu (P.P.); 3 School of Medicine, Josip Juraj Strossmayer University of Osijek, 31000 Osijek, Croatia; 4 Faculty of Dental Medicine and Health, Josip Juraj Strossmayer University of Osijek, 31000 Osijek, Croatia; 5 Eberly College of Science, The Pennsylvania State University, State College, PA 16802, USA; 6 School of Medicine, University of Split, 21000 Split, Croatia; 7 The Henry C. Lee College of Criminal Justice and Forensic Sciences, University of New Haven, New Haven, CT 06516, USA; 8 Sana Kliniken Oberfranken, 96450 Coburg, Germany; johannes.brachmann@regiomed-kliniken.de; 9 School of Medicine, University of Rijeka, 51000 Rijeka, Croatia; 10 School of Medicine, University of Pittsburgh, Pittsburgh, PA 15213, USA; 11 National Forensic Sciences University, Gandhinagar 382007, India; 12 Department of Molecular Biology, Faculty of Science, University of Zagreb, 10000 Zagreb, Croatia; 13 Department of Pediatrics, Clinical Hospital Center Rijeka, 51000 Rijeka, Croatia; 14 Department of Neuroscience, Yale School of Medicine, Yale University, 333 Cedar Street, New Haven, CT 06510, USA; martina.glavan@yale.edu (M.G.); ijoleru@gmail.com (I.O.); 15 Exogems SA, Biopole, 1066 Epalinges, Switzerland; pierre.rocheteau@gmail.com (P.R.); carlo.tremolada@gmail.com (C.T.); 16 Image Regenerative Clinic, 20122 Milano, Italy; 17 Restore Orthopedics and Sports Medicine, Sonora, CA 95370, USA; arianademers@gmail.com; 18 BioEvolve, San Diego Orthobiologics and Sports Center, San Diego, CA 92024, USA; info@bioevolvesports.com; 19 Texas Orthobiologics, Dallas, TX 75204, USA; info@biologicortho.com; 20 Department of Orthopaedics, UHealth Sports Medicine Institute, Miller School of Medicine, University of Miami, Miami, FL 33146, USA; dxk504@med.miami.edu (D.K.); cmc732@med.miami.edu (C.C.); dwk45@med.miami.edu (D.W.K.); ncp87@med.miami.edu (N.P.); 21 Cell Transplant Center, Diabetes Research Institute, Miller School of Medicine, University of Miami, Miami, FL 33146, USA; kbarbato@miami.edu; 22 FIRST Focused Integrated Regenerative Sports Medicine and Treatments, 8002 Zurich, Switzerland; pschnorr@first-zurich.ch; 23 Department of Immunology and Theranostics, Beckman Research Institute, City of Hope, Duarte, CA 91010, USA; 24 State Research Institute Center for Innovative Medicine, LT-08406 Vilniaus, Lithuania; raminta.vaiciuleviciute@imcentras.lt; 25 GaiaCell, Advanced Cell and Gene Therapy Ltd., Prevale 9, 1236 Trzin, Slovenia; zan.fortuna@gaiacell.net (Ž.F.); lara.opresnik@gaiacell.net (L.O.); blaz.curk@gaiacell.net (B.C.); miomir.knezevic@gaiacell.net (M.K.); gkz@gaiacell.net (G.K.Ž.); 26 Orthopaedic Clinic for Advanced Arthroscopic Sports and Regenerative Surgery Mitera Hospital, 15123 Athens, Greece; info@tsoukas-ortho.gr; 27 Department of Regenerative Medicine, State Research Institute Innovative Medicine Centre, LT-08406 Vilnius, Lithuania; ilona.uzieliene@imcentras.lt (I.U.); jolita.pachaleva@imcentras.lt (J.P.); eiva.bernotiene@imcentras.lt (E.B.); ali.mobasheri@oulu.fi (A.M.); 28 Dr. Phillip Frost Department of Dermatology and Cutaneous Surgery, University of Miami Miller School of Medicine, Miami, FL 33146, USA; iguanche@miami.edu (I.D.G.); ebadiavas@med.miami.edu (E.V.B.); 29 Medical Innovation Center (MEDIC), School of Medicine, European University Cyprus, Diogenis Str., Engomi, 2404 Nicosia, Cyprus; v.kalodimou@euc.ac.cy; 30 Centre Lémanique de Neuromodulation et Thérapie de la Douleur, Ensemble Hospitalier de la Côte (EHC), 1110 Morges, Switzerland; massimo.allegri@ehc.vd.ch; 31 Department of Orthopaedic Surgery, Yong Loo Lin School of Medicine, National University of Singapore, Singapore 119077, Singapore; 32 Faculty of Dentistry, National University of Singapore, Singapore 119077, Singapore; 33 Research Unit of Health Sciences and Technology, Faculty of Medicine, University of Oulu, 90570 Oulu, Finland; nancy.duartedelgado@oulu.fi; 34 Department of Joint Surgery, First Affiliated Hospital of Sun Yat-sen University, Guangzhou 510060, China; 35 Faculté de Médecine, Université de Liège, 4000 Liège, Belgium

**Keywords:** extracellular vesicles, exosomes, immune response, oncosomes, clinical translation, photobiomodulation

## Abstract

Extracellular vesicles (EVs) have emerged as essential mediators of intercellular communication, transporting a complex repertoire of lipids, proteins, and nucleic acids that mirror the physiological and pathological status of their parent cells. This review provides a comprehensive overview of EVs from their biogenesis and molecular composition to their translational potential in human disease. This review outlines the major classes of EVs, including exosomes, microvesicles, apoptotic bodies, and oncosomes, together with recent developments in their isolation, molecular characterization, and omics-based profiling. Special focus is given to the role of EVs in viral infection, inflammation, and immune regulation, as well as their contribution to disease development and cancer biology. Moreover, we highlight the emerging clinical applications of mesenchymal stem cell-derived EVs (MSC-EVs) in regenerative medicine and oncology, alongside the therapeutic modulation of EV signaling by photobiomodulation (PBM). Finally, we address key translational challenges related to standardization, scalability, and regulatory validation. As exosome-based therapeutics fall under strict FDA and EMA oversight, their translation further depends on harmonized quality controls and robust safety evaluation. By integrating molecular mechanisms with clinical applications, this review emphasizes the transformative potential of EVs as next-generation diagnostic and therapeutic tools in precision medicine.

## 1. Introduction

Extracellular vesicles (EVs) are nanoscale, membrane-enclosed particles actively released by a wide range of cell types, carrying targeted biologically active molecules [1,2,3]. They encapsulate a diverse repertoire of bioactive molecules, including lipids, proteins, nucleic acids, and metabolites, reflecting the physiological or pathological state of their cell of origin [2,4]. Upon uptake by recipient cells, EVs deliver this molecular cargo, thereby modulating cellular function and enabling highly specific intercellular communication [2]. Through these mechanisms, EVs have emerged as key mediators in diverse biological processes such as immune regulation, tumor progression, viral pathogenesis, and the pathophysiology of neurological disorders.

**Figure 1:**
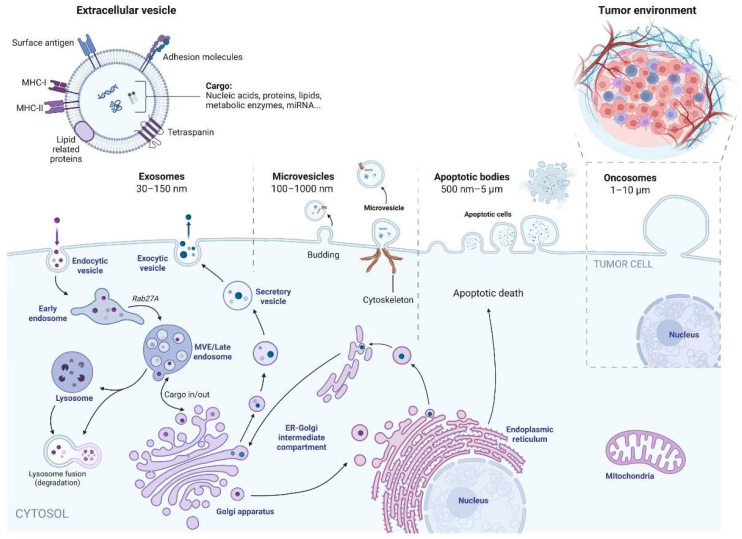
Biogenesis and classification of extracellular vesicles (EVs). The schematic illustrates the intracellular pathways involved in EV formation and release in a normal cell and within a tumor cell and its microenvironment. Key cellular compartments are depicted, including the nucleus, mitochondria, endoplasmic reticulum (ER), Golgi apparatus, ER-Golgi intermediate compartment, early and late endosomes, multivesicular endosomes (MVEs), lysosomes, and secretory vesicles. Exosomes (30–150 nm) originate from multivesicular endosomes (MVEs) formed through the endosomal pathway, which can either fuse with lysosomes for degradation or with the plasma membrane to release exosomes into the extracellular space. Microvesicles (100–1000 nm) arise via direct budding from the plasma membrane. Apoptotic bodies (500 nm–5 μm) are released from apoptotic cells, while larger oncosomes (1–10 μm) are shed from tumor cells. EVs carry diverse cargo, including nucleic acids, proteins, lipids, metabolic enzymes, and microRNAs, and surface markers such as MHC-I, MHC-II, tetraspanins, lipid-related proteins, adhesion molecules, and antigens. Regulatory proteins like Rab27A are involved in MVE trafficking. The tumor environment is shown with EVs interacting in the extracellular space, highlighting their role in intercellular communication (Created in BioRender. https://BioRender.com/jqlgyj8, accessed on 15 December 2025).

There are various types of EVs that differ in their cellular origin and biogenesis. These include endosome-origin “exosomes”, plasma-membrane-derived microvesicles (also referred to as ectosomes or microparticles), apoptotic bodies released during programmed cell death, and more specialized populations such as oncosomes (Figure 1). Each EV subtype contributes uniquely to intercellular communication, with distinct implications for physiological regulation, disease progression, and emerging therapeutic applications. Their classification is primarily based on their site of origin and mode of biogenesis, which underlie their molecular composition and functional properties [2,4].

Exosomes, which originate from multivesicular bodies and typically measure 30–150 nm, are increasingly harnessed as therapeutic tools, particularly in neurology, where their ability to cross the blood–brain barrier (BBB) enables targeted delivery of bioactive molecules [1,5,6]. Their BBB-crossing capability occurs via mechanisms like receptor-mediated transcytosis or endocytosis, making them promising for treating conditions such as Alzheimer’s and Parkinson’s in ongoing preclinical and Phase I/II clinical trials [7,8]. Exosomes are being actively explored in preclinical and clinical settings, with growing interest in their potential applications across adipose tissue, skeletal muscle, and the heart [1,9]. Although these areas have traditionally posed biological challenges such as tissue-specific barriers and lower vascular permeability, advances in delivery strategies and engineering approaches are increasingly opening the door to broader therapeutic use.

In contrast to exosomes, plasma membrane-derived microvesicles arise through outward budding and fission of the cell surface and typically range from 100 to 1000 nm in size [10]. In erythrocytes, this process contributes to vesicle release into the extracellular milieu during erythropoiesis, cellular senescence, and under pathological or stress conditions [11,12,13]. At a wider biological scale, plasma membrane-derived microvesicles have emerged as informative biomarkers in metabolic and hematologic disorders, including diabetes (e.g., elevated levels correlating with complications like retinopathy, neuropathy and thrombosis) and various blood diseases [12,14,15]. Beyond mammals, microvesicles are also secreted across unicellular organisms (e.g., as outer membrane vesicles in bacteria for nutrient exchange and defense), and in tumors they are abundantly produced and have been investigated as platforms for engineered cancer vaccines due to their immunogenicity and ability to carry tumor antigens [16,17].

Apoptotic bodies are EVs generated during programmed cell death [18,19]. Due to their origin, they are closely linked to immune cell-associated processes and pathologies, such as promoting efferocytosis (phagocytic clearance) to prevent inflammation and autoimmunity [20]. In addition, their comparatively larger size (typically 500 nm–5 μm) and stable circulation allow them to transport a greater repertoire of functional biomolecules [21,22].

Oncosomes represent a distinct class of EVs associated with oncogenic transformation and highly metastatic behavior [23,24]. They are typically larger (1–10 μm) and derived from aggressive tumor cells via membrane blebbing [25]. Tumor-derived EVs, including oncosomes, carry molecular signatures of their malignant origin (e.g., mutant DNA, miRNAs, and proteins like EGFR) and are being investigated as tools for more precise early cancer diagnosis through liquid biopsies with high sensitivity for detecting metastasis [26,27]. Compared with conventional blood markers, EVs offer additional diagnostic value by encapsulating tumor-derived nucleic acids and proteins that reflect the molecular status of the tumor and remain stable in circulation. This enhanced molecular information supports the use of EVs as sensitive biomarkers for early cancer detection [28,29]. More broadly, the diverse biological activities of EVs and their central role in intercellular communication underpin their growing clinical relevance for disease diagnosis, prognosis, and therapy [30,31].

## 2. Origin and Molecular Characterization of EVs

Exosomes, among the various EV subtypes, are the most extensively characterized and have been the focus of intense investigation due to their unique biogenesis pathway and versatility in mediating intercellular communication [32]. The context of their biogenesis critically shapes their molecular composition, thereby influencing delivery, uptake, and functional cargo in recipient cells [33]. Thus, it is important to understand exosome biology to decipher their diverse roles across physiological and pathological settings.

Once secreted, exosomes engage with recipient cells through a combination of targeting, internalization, and secondary release mechanisms [33]. These interactions enable exosomes to mediate both local and long-distance intercellular communication [33]. Once exosomes reach recipient cells, they exert their effects through three principal mechanisms: internalization, direct membrane fusion, or receptor–ligand interactions [33]. Following internalization, exosome-containing endosomes mature into MVB and are ultimately degraded by lysosomes within the target cell [33]. The efficiency and mode of uptake vary depending on the exosome’s origin [34]. Intraspecies exosomes are preferentially internalized by their parental cells through receptor–ligand interaction-mediated endocytosis, often clathrin-dependent, with surface ligands like integrins enhancing uptake efficiency by 1.4–3.2 times compared to cross-species exosomes [34]. By contrast, cross-species exosomes, despite lower uptake efficiency, are predominantly internalized by non-parental cells via direct membrane fusion [34]. Such differences highlight evolutionary adaptations in exosome communication, with intraspecies interactions favoring targeted signaling and cross-species ones enabling broader interkingdom exchanges, such as in microbial-host interactions [35].

Evidence suggests that exosome–tissue binding sites share strong similarities between certain organs, such as the brain and lung or the spine and liver [36]. For instance, integrins like α6β4 and α6β1 on exosomes promote tropism to lung and brain, while β1 variants target liver and bone (spine-related skeletal sites) [37]. This selective targeting contributes to metastasis patterns, as tumor-derived exosomes condition these organs in ways that support subsequent cancer cell colonization. Similarly, exosomes not matched by origin (e.g., mouse versus human; cancer versus non-cancer) may converge on a common set of binding interactions with recipient tissues [36]. However, exosomes derived from different cellular sources exhibit distinct molecular compositions and functions, underscoring the importance of considering the characteristics of the parent cell when evaluating their biological or therapeutic potential [38].

The molecular composition of exosomes, driven by their cellular origin, includes a lipid bilayer rich in cholesterol, sphingolipids, ceramide, and phospholipids, and carries a diverse molecular cargo that includes proteins such as tetraspanins (e.g., CD9, CD63, CD81) [39,40], heat shock proteins, and signaling molecules, as well as nucleic acids including mRNA fragments and microRNAs (Figure 1) [41]. This composition enables exosomes to modulate gene expression and cellular behavior, making them critical in disease pathophysiology and promising for diagnostic and therapeutic applications [41].

Exosomes have emerged as critical players in disease pathophysiology and hold considerable promise as diagnostic and therapeutic tools, with applications already explored in maternal health [42], oncology [43], and skeletal disorders [44]. For instance, placental exosomes, secreted by placental cells throughout pregnancy, carry pro-proliferative, pro-anabolic, anti-catabolic, and anti-inflammatory proteins, positioning them as potential biomarkers for maternal disorders like preeclampsia, gestational diabetes mellitus (GDM), and fetal growth restriction [42,45,46]. Beyond physiological contexts, exosomes also participate in pathological processes, particularly in cancer. Exosomes from cancer cells influence metastasis by exhibiting tropism to specific organs (e.g., α6β4 and α6β1 integrins targeting lung and brain, β1 variants targeting liver and bone) [37].

Biological fluids contain exosomes secreted by a wide range of tissues [47]. The heterogeneous molecular cargo of exosomes in these fluids under both physiological and pathological conditions supports their use as minimally invasive biomarkers, particularly in cancer detection and monitoring [48]. Variations in exosome composition and circulating levels in response to diverse stimuli influence disease progression in skeletal muscle, highlighting their potential utility in musculoskeletal research and clinical applications [44], including stem cell-derived exosomes for promoting myogenesis and reducing atrophy in conditions like sarcopenia [49].

## 3. Cellular Sources for Isolation of Exosomes

The ability to isolate exosomes from diverse biological matrices is pivotal for their application in research and clinical diagnostics, as the choice of source influences accessibility, yield, and relevance to specific disease contexts. These matrices, which include bodily fluids and cell culture derivatives, provide practical avenues for obtaining exosomes tailored to translational needs.

Blood-derived sources, such as plasma and serum, are among the most commonly used due to their accessibility through routine venipuncture and their relevance in systemic conditions like cancer, cardiovascular diseases, and inflammatory disorders [50]. Exosomes can also be isolated from blood cells, including platelets, erythrocytes, and leukocytes, which release vesicles in response to physiological or pathological stimuli, offering insights into processes like hemostasis or immune activation [51].

Urine provides a non-invasive source of exosomes, primarily originating from the kidneys and urinary tract, making it ideal for studying renal disorders, urological cancers, or systemic conditions reflected in excreted vesicles [52]. Cerebrospinal fluid (CSF), although obtained invasively via lumbar puncture, is a valuable source for central nervous system-derived exosomes, critical for investigating neurodegenerative diseases such as Alzheimer’s or multiple sclerosis, where brain-specific biomarkers are detected [53].

Additional biological matrices, such as saliva, breast milk, ascites fluid, synovial fluid, and amniotic fluid, offer unique opportunities for specific applications. For instance, salivary exosomes are promising for oral cancer diagnostics [54], while breast milk exosomes may inform neonatal health studies [55]. Cell culture supernatants from in vitro models allow controlled exosome isolation from specific cell types, facilitating mechanistic studies prior to clinical translation [56].

The choice of source influences isolation techniques, such as ultracentrifugation, precipitation, or immunoaffinity capture, and downstream applications. By leveraging these accessible and disease-relevant matrices, clinicians can advance exosome-based diagnostics and personalized medicine.

## 4. Structure and Molecular Composition (Omics Characterization; Lipidomics, Proteomics, Transcriptomics)

Proteins are central to exosome structure and function, residing either within the lipid bilayer or in the hydrophilic core [57]. They play key roles in mediating adhesion, recognition, and uptake by recipient cells. Canonical exosomal proteins include members of the tetraspanin family (such as cluster of differentiation 9 (CD9/TSPAN29), cluster of differentiation 63 (CD63/LAMP-3/TSPAN30), and cluster of differentiation 81 (CD81/TAPA-1/TSPAN28)), epithelial cell adhesion molecule (EpCAM), integrins, and other adhesion-related molecules, many of which serve as widely used exosomal markers [54]. These proteins not only contribute to exosome biogenesis and trafficking but are also implicated in diverse disease processes, including cancer, neurodegenerative diseases, and immune responses [50].

Exosome biogenesis involves the maturation of early endosomes into MVBs, a process classically governed by the endosomal sorting complex required for transport (ESCRT) machinery [58]. ESCRT complexes regulate membrane budding, intraluminal vesicle formation, and MVB development [59]. In addition, ESCRT-independent pathways also contribute to exosome biogenesis, often relying on lipid-driven mechanisms (e.g., ceramide) and tetraspanins to facilitate cargo loading and vesicle formation [58,60]. Ceramide, produced by neutral sphingomyelinase (n-SMase), induces negative membrane curvature, promoting the formation of intraluminal vesicles (ILV), the precursors of exosomes. Interestingly, under specific conditions, membrane-associated histone proteins can be released via the MVB–exosome pathway, and under conditions of cellular stress, histone upregulation has been associated with the production of smaller exosomes [61,62]. Once secreted into extracellular fluids, exosomes do not exist as isolated vesicles but rapidly acquire a dynamic layer of proteins, lipids, and other biomolecules derived from their environment, termed the protein corona [63]. This corona further influences exosome stability, biodistribution, uptake, and ultimately their biological and therapeutic activity.

Exosomes can encapsulate endogenous miRNAs, which regulate diverse physiological and pathological processes, including apoptosis of cancer cells, lipid metabolism, and angiogenesis [64]. The selective incorporation of miRNAs into exosomes is mediated by both ESCRT-dependent and ESCRT-independent pathways, involving specific RNA-binding proteins (e.g., hnRNPA2B1, YBX1, and AGO2) that recognize sequence motifs or structural features. Exosomal miRNAs have attracted particular attention as minimally invasive biomarkers of cancer, given their stability in circulation and ability to reflect the molecular state of the tumor [65].

Exosomes also play an important role in regulating lipid metabolism, a process that strongly contributes to the establishment of the tumor microenvironment, as well as to tumor cell invasion, migration, and chemoresistance [66]. One key mechanism involves the transport of lipid-metabolizing enzymes and receptors via exosomes, thereby influencing lipid homeostasis within the tumor milieu. n-SMase is a central regulator of lipid metabolism in the ESCRT-independent pathway, where it catalyzes the hydrolysis of sphingomyelins (SMs) into ceramides (Cers), promoting the inward budding of the endosomal membrane and the formation of intraluminal vesicles within MVBs [67]. Modulation of n-SMase activity directly impacts exosome biogenesis and secretion, and pharmacologic inhibition of n-SMase has been shown to reduce exosome release, highlighting its relevance as a potential therapeutic target in cancer [64]. Moreover, exosomal lipids, such as cholesterol and phosphatidylserine, contribute to membrane rigidity and signaling, further influencing tumor progression [68].

## 5. Biogenesis and Biological Functions

**Figure 2:**
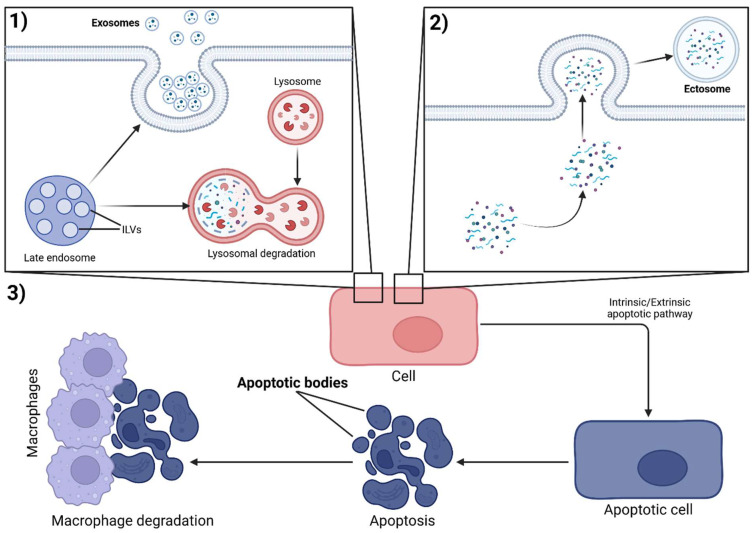
Different biogenic pathways of extracellular vesicles pertaining to: (**1**) exosomes, (**2**) ectosomes (microvesicles), and (**3**) apoptotic bodies (created with Biorender.com); ILV—intraluminal vesicles (Created in BioRender. https://BioRender.com/wln1nyc, accessed on 15 December 2025).

The biogenesis of extracellular vesicles takes place through three different pathways, depending on whether they are classified as exosomes, ectosomes (microvesicles) or apoptotic bodies (Figure 2) [69].

The process of exosome biogenesis is primarily tied to intraluminal vesicles (ILV), formed by the invagination of late endosome membranes. While a smaller percentage of ILVs experience degradation by lysosomes, most are ejected into the extracellular space, at which point they become exosomes. Constitutively, exosomes comprise over four thousand proteins and over two thousand RNAs [70]. In the context of exosome biogenesis, two crucial pathways must be addressed. The Endosomal Sorting Complex Required for Transport (ESCRT)-dependent pathway is mediated by several protein complexes (ESCRT-0, ESCRT-I, and ESCRT-II), which actively facilitate the movement of cargo related to endosomes. This cargo recognition and subsequent sorting process involves members of the Cbl family of E3 ubiquitin ligases. On a cellular level, the ESCRT-dependent pathway maintains integrity and function through membrane repair, protein degradation, and exosome formation [71]. Additionally, the formation of exosomes is facilitated by the ESCRT-independent pathway, which refers to mechanisms of exosome biogenesis that do not rely on ESCRT machinery, but instead involve, tetraspanins (e.g., CD9, CD63, CD81), lipid-driven mechanisms, such as ceramide formation via neutral sphingomyelinase 2 (nSMase2) and cholesterol, heat shock proteins, and other scaffolding molecules. This pathway is essential for expanding cellular functionality, primarily through cellular communication [72].

Unlike exosomes, the biogenesis of ectosomes is primarily tied to the budding of plasma membranes. Once detached from the membrane, ectosomes are most commonly released into the circulation, with platelet ectosomes being the most prominent example [73]. Finally, a third mechanism of biogenesis is tied to apoptotic bodies (ApoBD). Unlike the previous two mechanisms, which involve controlled formation and release, ApoBDs are formed after the cellular decomposition of an apoptotic cell. After their formation, ApoBDs are usually degraded by macrophages and parenchymal cells, without the occurrence of an inflammatory reaction [19].

Once in the extracellular space, EVs can use their cargo to influence several cellular and biomolecular processes. Primarily, this depends on the type of cell that is the source of the vesicle. One of these processes is proliferation, which has been the focus of not only cellular research but also cancer research. For example, previous studies have established that vesicles carrying specific RNA-type cargo can enhance the proliferation of tumor cells in hepatocellular carcinoma and laryngeal squamous cell carcinoma [74]. Secondly, it is well-known that the contents of EVs may stimulate or suppress the immune response. This is achieved via presentation of foreign antigens or the release of proinflammatory or anti-inflammatory mediators [75]. Thirdly, multiple studies have demonstrated the regenerative properties of EVs, investigating the regeneration of tendons, skeletal muscles, and even peripheral nerves [76]. Alongside these key aspects, EVs mediate other processes via intracellular communications as messengers, as well as transcellular biomolecule transfer involving proteins, lipids, metabolites and nucleic acids (mRNA and microRNA) [77].

## 6. Roles in Viral Infection

### 6.1. Exosomes as Drivers of Viral Infection

Viruses are tiny, simple non-cellular organisms that cannot survive outside of living cells. An increasing number of studies have found a close relationship between viruses and exosomes. For instance, retroviruses can utilize vesicle production mechanisms to promote budding, while hepatitis viruses can use exosomes for cell-to-cell transmission [78,79]. Exosomes, nanoscale EVs secreted by nearly all cell types, play a dualistic and nuanced role during viral infections. While they are integral to intercellular communication and immune modulation, viruses have evolved mechanisms to exploit exosomes to enhance their own propagation, evade immune detection, and modify the host microenvironment.

A prominent example is HIV. Exosomes derived from HIV-infected cells are enriched in viral components such as Nef protein, Gag, TAR-RNA, and specific miRNAs [80]. These exosomes promote HIV pathogenesis through multiple mechanisms: Nef-loaded exosomes induce apoptosis in CD4+ T cells, reduce CD4 expression on recipient cells, and modulate the expression of co-receptors such as CCR5, thus facilitating further infection. They also inhibit apoptosis in infected or bystander cells by downregulating pro-apoptotic factors like Bim and Cdk9 [81]. However, although HIV evolved to exploit exosomes to expand and escape the immune system, those exosomes are also important for anti-viral immunity. Exosomes in healthy human semen block the transmission of HIV-1 from vaginal epithelial cells to target cells and inhibit HIV from crossing the vaginal epithelial barrier in vitro [82]. Also, after the internalization of exosomes, they exert an antiviral response by blocking the activity of HIV reverse transcriptase and reduce the replication of the viral complex [83].

In hepatotropic viruses such as HBV, studies have found that exosomes released by HBV-infected cells contain intact viral particles [84]. This is important because there are an estimated of 257 to 270 million HBV carriers worldwide, with a prevalence of chronic HBV infection of 3.5% [85]. Exosomes mediate viral RNA and protein transfer between hepatocytes, promoting antibody-independent viral spread while evading the immune system. HBV-infected hepatocytes release exosomes containing viral DNA, HBsAg, HBeAg, and immunosuppressive miRNAs (e.g., miR-21, miR-192, miR-215), which dampen the host immune response by targeting IL-21 or promoting PD-L1 expression on monocytes [86]. Like previously when the contents of exosomes are viral proteins, nucleic acids, and other substances, exosomes promote HBV diffusion. When the contents are anti-HBV factors, exosomes have the effect of inhibiting viral activity and antiviral infection. And the cargos also act as a ligand of immune cells to activate antiviral immunity.

SARS-CoV-2, the causative agent of COVID-19, relies on spike protein (S protein) binding to angiotensin-converting enzyme 2 (ACE2) on the cell membrane. After the spike protein binds to ACE2, TMPRSS2 cleaves the spike protein at the cell surface, a step that activates the fusion process and allows the virus to enter the cell through a membrane fusion pore [87]. On the one hand this is exploited with exosomes for viral dissemination, as exosomes released from infected cells may carry ACE2 receptors and viral RNA, facilitating the spread of the virus to distant, otherwise less-permissive tissues [88]. On the other hand, these vesicles expressing ACE2 have specific exosome markers, neutralizing SARS-CoV-2 by competitive binding to ACE2, with the same or higher inhibitory effect. At the same time, it can reduce the mortality of SARS-CoV-2 infection in mouse models [89]. Of note, SARS-CoV-2 infection alters the proteomic profile of circulating exosomes, enriching them in proteins associated with inflammation, coagulation, and immune modulation, particularly in severe that can be part of what leads to sepsis-like disease characterized by decreasing lymphocytic count and cytokine storm [90].

### 6.2. Exosomes in Viral Detection

Exosomes can also serve as non-invasive biomarkers for the detection and monitoring of viral infections. Their stability in biological fluids and enrichment in viral or disease-associated components make them attractive for liquid biopsy applications. In HIV, exosomes isolated from semen, blood, or cerebrospinal fluid contain detectable levels of Nef mRNA, TAR-RNA, and miR-155. These markers not only reflect viral load but are also associated with HIV-associated neurocognitive disorders [86]. In HBV, miRNAs such as miR-122 and HBV-miR-3 carried by exosomes correlate with disease progression, fibrosis stage, and liver inflammation. For instance, miR-122 is significantly elevated in early infection but decreases in advanced fibrosis, offering a dynamic readout of liver pathology allowing the adaptation of clinical management [81].

For COVID-19, exosomes have emerged as a promising platform for disease detection and severity assessment. Numerous studies have demonstrated that SARS-CoV-2 infection induces a distinct alteration in the cargo of circulating exosomes [91]. Proteomic analyses of plasma-derived exosomes from COVID-19 patients have identified elevated levels of C-reactive protein (CRP), alpha-1-acid glycoproteins, complement proteins, and pro-coagulant molecules such as CD142. These biomarkers are strongly associated with disease severity and could serve as prognostic indicators [91,92].

Furthermore, exosomes can harbor SARS-CoV-2 RNA fragments, including those from the spike, nucleocapsid, and envelope genes, enabling direct detection of viral presence in patient biofluids. This is particularly valuable for detecting infection in hard-to-access tissues or for cases where nasopharyngeal swabs yield false negatives. In that context it was demonstrated that exosomes from infected lung epithelial cells transferred viral RNA to cardiomyocytes and endothelial cells in vitro, suggesting a role in systemic viral dissemination and providing a rationale for liquid biopsy diagnostics [93].

Exosomal analysis has also been proposed for monitoring treatment response and recovery. Longitudinal profiling of patients recovering from COVID-19 showed dynamic changes in exosomal protein and RNA content, including normalization of inflammatory markers and downregulation of pro-thrombotic factors. In addition, exosomal signatures may aid in identifying individuals at risk for long COVID by capturing persistent alterations in immune-related proteins and cytokine signaling pathways [94,95].

The potential for exosome-based multiplexed diagnostics using microfluidic platforms or immunoaffinity assays is currently under development. These approaches will allow for rapid, non-invasive, and high-throughput screening of SARS-CoV-2 infection and progression using small volumes of blood, saliva, or urine.

### 6.3. Exosomes as Antiviral Therapeutics

Beyond their role in pathogenesis and diagnostics, exosomes are emerging as promising therapeutic tools, either as natural antiviral agents or as engineered delivery vehicles. Natural antiviral exosomes have been identified in human fluids. Seminal and breast milk-derived exosomes can inhibit HIV transmission by blocking reverse transcriptase activity and interfering with viral entry [96]. Breast milk exosomes, for example, compete with HIV for DC-SIGN receptors on dendritic cells, reducing the risk of vertical transmission [83]. Similarly, exosomes derived from IFN-stimulated epithelial cells carry antiviral factors that inhibit HIV replication in macrophages via the induction of (interferon-stimulated genes) ISGs and APOBEC3G [97].

Engineered exosomes are under clinical and preclinical development. For example, as therapy for COVID-19, mesenchymal stem cell (MSC)-derived exosomes have been tested in multiple clinical trials for patients with severe pneumonia. These exosomes exhibit immunomodulatory properties, reduce neutrophil counts, and improve oxygenation [91]. Exosomes have been functionalized with ACE2 to act as decoys or inhibitors, respectively, against SARS-CoV-2. ACE2-expressing exosomes, often termed “nano-decoys,” mimic the surface of host cells and bind the viral spike protein, thereby neutralizing SARS-CoV-2 before it can enter epithelial cells. Studies have demonstrated that these ACE2-decorated vesicles effectively reduce viral load in vitro and in animal models [98,99]. On the other hand, exosomes engineered to carry interferon-induced transmembrane protein 3 (IFITM3) exhibit intrinsic antiviral activity by restricting viral membrane fusion. This approach benefits from the endosomal origin of exosomes and the enrichment of antiviral host factors in their membrane [100]. These strategies leverage the natural biocompatibility and tissue-targeting properties of exosomes to deliver precise and potent antiviral effects with minimal off-target consequences.

Exosomes also serve as new and more efficient vectors for vaccines that are being explored using viral antigens, such as the SARS-CoV-2 spike protein, or viral mRNA packaged into exosomal membranes. These exosomes can be naturally derived or engineered to display antigenic peptides or encapsulate nucleic acid cargo. Compared to traditional viral vectors or lipid nanoparticles, exosome-based platforms offer several advantages: enhanced tissue tropism due to intrinsic targeting properties, increased stability at room temperature, and reduced risk of triggering excessive innate immune responses. Preclinical studies have shown that exosomes decorated with SARS-CoV-2 spike protein induce strong humoral and cellular immunity, including neutralizing antibodies and T-cell responses, in vaccinated animals. mRNA-loaded exosomes, delivered via intramuscular injection, have also demonstrated immunogenicity while exhibiting superior biocompatibility and biodistribution profiles compared to lipid-based carriers. In addition, exosome platforms are being tested for mucosal delivery routes, including intranasal administration, to induce both systemic and local immunity. As such, exosome-based vaccine systems are being positioned as promising next-generation tools for pandemic preparedness and immunotherapy [93,101,102].

Taken together, the growing body of evidence emphasizes the multifaceted roles of exosomes in viral infections. They act as potent mediators of viral pathogenesis, facilitating viral dissemination, modulating host immunity and promoting immune evasion. At the same time, the cargo of exosomes reflects the physiological or pathological state of the cells from which they originate, making them invaluable as non-invasive biomarkers for detecting infection, monitoring disease progression and stratifying patient risk. Furthermore, both naturally occurring and engineered exosomes offer distinct advantages for therapeutic applications, ranging from the inhibition of viral replication to the delivery of vaccines and antiviral agents with high specificity and minimal toxicity. As research advances, exosomes are emerging not just as passive bystanders, but as active participants and tools in our fight against viral diseases. Their biological plasticity and translational potential warrant continued exploration to unlock new frontiers in virology, diagnostics, and immunotherapy.

## 7. Microbial EVs at the Crossroad of Inflammation and Immunity

Microbial EVs, particularly those derived from bacteria (bEVs), have emerged as pivotal mediators in host-microbe interactions. Secreted by both Gram-positive and Gram-negative bacteria, these membrane-bound nanostructures carry bioactive molecules, including proteins, lipids, nucleic acids, and metabolites that can influence cellular pathways across species barriers. Increasingly, bEVs are recognized as active players in inflammation, capable of either promoting immune activation or contributing to immune tolerance and homeostasis, depending on their origin and context [103,104].

### 7.1. Biogenesis and Structural Diversity of Microbial EVs

The biogenesis of bacterial extracellular vesicles (bEVs) reflects the structural complexity and diversity of bacterial cell envelopes. In Gram-negative bacteria, EV formation can occur through blebbing of the outer membrane, generating outer membrane vesicles (OMVs) [105]. This process encapsulates periplasmic components and outer membrane-derived molecules such as lipopolysaccharides (LPS), outer membrane proteins, phospholipids, and virulence factors, which confer strong immunostimulatory potential to OMVs [106]. Variations in environmental conditions, including stress and nutrient availability, can modulate the composition and release rates of OMVs, contributing to bacterial adaptation and pathogenicity [107]. Recent studies have identified novel mechanisms such as explosive cell lysis, outer membrane remodeling through lipid A diacylation, and involvement of a highly conserved phospholipid transporter (VacJ/Yrb ABC) in OMV formation [108]. VacJ and Yrb are proposed to uphold lipid asymmetry in the outer membrane (OM) of Gram-negative bacteria. This is accomplished by the reverse translocation of phospholipids from the OM to the inner membrane (IM). Thus, Gram-negative bacterial vesicle subtypes are the result of distinct formation pathways. OMVs are formed through the disruption of the outer membrane and peptidoglycan cell wall, leading to membrane budding. In contrast, explosive outer-inner membrane vesicles and outer-inner membrane vesicles are generated via explosive cell lysis. Explosive outer-inner membrane vesicles are only composed of the outer membrane, while outer-inner membrane vesicles possess both outer and inner membrane bilayers [109]. Both types of microvesicles contain DNA, RNA, virulence factors, cytoplasmic and membrane proteins and sometimes bacteriophages [109].

Gram-positive bacteria, initially thought incapable of vesicle production due to thick peptidoglycan cell wall and lack of an outer membrane, are now known to release membrane vesicles. These vesicles emerge from the cytoplasmic membrane via local weakening or enzymatic remodeling of the peptidoglycan layer, often involving endolysins or autolysins [109]. Stress responses, antibiotic exposure, and growth phase can further influence microvesicle production. These microvesicles encapsulate cytoplasmic and membrane proteins, nucleic acids, and lipoteichoic acids, which can mediate potent immune responses via Toll-like receptor 2 (TLR2) [110].

EVs from both Gram-negative and Gram-positive bacteria typically range from 20 to 400 nm in diameter and display significant heterogeneity in morphology, cargo, and functional activity, even within vesicles secreted by a single bacterial strain. This diversity arises from distinct biogenesis pathways, selective cargo sorting, and environmental cues, positioning microbial EVs as highly adaptable vehicles of intercellular communication [111].

### 7.2. Microbial EVs as Triggers and Modulators of Inflammation

The bEVs represent a central axis in the modulation of inflammatory processes, exerting pro- and anti-inflammatory effects depending on their microbial origin, structural composition, and interaction with host immune pathways. Their immunogenic potential stems from the diverse array of bioactive molecules they carry, including pathogen-associated molecular patterns (PAMPs) such as lipopolysaccharides (LPS), peptidoglycans, lipoproteins, flagellin, and bacterial DNA or RNA27 [103].

Pathogenic Gram-negative bacteria such as *Escherichia coli*, *Helicobacter pylori*, and *Pseudomonas aeruginosa* produce OMVs rich in LPS, outer membrane proteins, and virulence factors. These vesicles engage Toll-like receptors (TLRs), particularly TLR4 and TLR2, leading to activation of NF-κB and mitogen-activated protein kinase (MAPK) pathways. This cascade triggers the production of pro-inflammatory cytokines including TNF-α, IL-1β, IL-6, and IL-8, promoting neutrophil recruitment, endothelial activation, and epithelial barrier dysfunction [112,113]. In some contexts, these vesicles can also enhance inflammasome activation and pyroptotic cell death, exacerbating tissue injury and disease pathology [103].

In contrast, bEVs derived from commensal or probiotic strains such as Lactobacillus plantarum, Bifidobacterium longum, or Akkermansia muciniphila often exert immunoregulatory effects. These vesicles can modulate the immune response by enhancing the production of anti-inflammatory cytokines like IL-10 and TGF-β, promoting regulatory T cell (Treg) differentiation and suppressing Th1/Th17-mediated responses [103,107]. Through interactions with intestinal epithelial cells and antigen-presenting cells, they can also contribute to mucosal tolerance and maintenance of gut homeostasis [114].

Recent studies also suggest that bEVs can modulate the systemic immune response. For example, OMVs in circulation can prime innate immune cells, a phenomenon termed trained immunity or induce tolerance through epigenetic reprogramming of monocytes and macrophages. This underlies their potential involvement in chronic inflammatory conditions, including metabolic syndrome, atherosclerosis, and neuroinflammation [115].

Overall, microbial EVs function as context-dependent immunomodulators. Their pro-inflammatory or tolerogenic capacity is not fixed but varies according to microbial species, environmental stressors, and host immune status. This intricate interplay positions bEVs as both biomarkers and therapeutic agents in inflammation-centric diseases, warranting further investigation into their diagnostic and clinical utility.

### 7.3. Crosstalk Between Microbial EVs and Host Immunity

Beyond triggering innate immunity, bEVs profoundly influence the adaptive immune system, serving as potent modulators of antigen presentation, lymphocyte activation, and immune homeostasis [109]. bEVs can function as carriers of immunogenic peptides and microbial antigens, which are delivered to dendritic cells and other antigen-presenting cells (APCs). These antigens are then processed and presented via major histocompatibility complex (MHC) class I and II molecules, leading to the activation of CD8+ cytotoxic T cells and CD4+ helper T cells, respectively [109]. In particular, OMVs from Gram-negative bacteria such as Legionella pneumophila and Salmonella enterica have been shown to upregulate MHC expression and co-stimulatory molecules (CD80/CD86) on dendritic cells, enhancing their antigen-presenting capacity or inducing macrophages to produce pro-inflammatory cytokines, thereby amplifying the local inflammatory milieu [116]. Neisseria meningitidis bEVs have shown to elicit the secretion of tumor necrosis factor alpha (TNFα) and interleukin-1β (IL-1β) from neutrophils [117]. Staphylococcus aureus-derived bEVs activates the NLR family pyrin domain containing 3 (NLRP3) inflammasome but also the IL-1β, IL-18, and caspase-1 activity of the host and the pathogen Tannerella forsythia derived OMVs induced proinflammatory responses via TLR2 activation [118,119].

Furthermore, bEVs contribute to shaping the cytokine environment that is key for immune response. Depending on their molecular content and microbial origin, bEVs can either promote inflammatory responses like previously described or foster regulatory immune pathways like bEVs originating from *Helicobacter pylori* that have been identified as inducer of the immunosuppressive cytokine IL-10 from human peripheral blood monocytes promoting an anti-inflammatory milieu [120].

bEVs are also implicated in immune evasion strategies. Certain vesicles can carry proteases or lipids that inhibit phagosome-lysosome fusion, suppress oxidative bursts in phagocytes, or interfere with antigen processing [121]. Others contain small RNAs or peptidoglycan derivatives that manipulate host cell signaling to dampen pro-inflammatory responses [121]. For example, vesicles from Staphylococcus aureus have been reported to affect host cells that contribute to bacterial clearance, with toxin family in the bEVs playing a critical role in immune evasion, although many reports show that bEVs from this bacterium promote immune reaction (cf upper part) [122]. Additionally, recent evidence suggests that bEVs may prime the host’s immune system for long-term responses. By delivering microbial components to lymphoid organs, they can establish a state of trained immunity or tolerance, potentially influencing vaccine responses and susceptibility to chronic inflammatory or autoimmune conditions [123].

Collectively, these findings highlight the nuanced and multifaceted interactions between microbial bEVs and the host immune system, positioning bEVs as critical regulators of immune surveillance, tolerance, and disease outcomes.

### 7.4. Therapeutic and Diagnostic Potential

Given their nanoscale size, membrane structure, and innate ability to carry complex biological cargo, microbial EVs have garnered considerable attention as tools in therapeutics and diagnostics. Their ability to cross biological barriers, such as the blood-brain and intestinal epithelial barriers, facilitates systemic delivery of their contents, including proteins, lipids, metabolites, and nucleic acids. This makes them ideal candidates for the development of next-generation delivery vehicles in biomedicine.

Engineered bEVs, particularly outer membrane vesicles (OMVs) from Gram-negative bacteria, are being actively explored as platforms for vaccine development. These vesicles can be modified to display antigens from pathogenic bacteria or viruses, thereby serving as immunogenic, self-adjuvanted nanoparticle vaccines. Examples include OMV-based vaccines for Neisseria meningitidis (NCT04722003) and experimental formulations against pathogens like Salmonella, Shigella, and even SARS-CoV-2 (NCT05604690). Their immunogenicity, scalability, and natural adjuvant properties (like LPS or lipoproteins) make them attractive over synthetic nanocarriers [104,124]. Moreover, bEVs are being evaluated as therapeutic agents in cancer and regenerative medicine. It was demonstrated that the administration of OMVs derived from *Escherichia coli* led to complete remission of MB49 and EMT6 tumors in most treated mice [125]. *Escherichia coli* OMV treatment significantly enhanced the infiltration of both total CD3+ T cells and CD8+ T cells into the tumor tissue in the MC38-OVA tumor model. In the field of regenerative medicine bEVs have shown very potent results in bone regeneration thanks to their inner property but also for their capacity to be loaded with many molecules to boost regeneration. For example, it was demonstrated that OMVs from the Gram-negative bacterium Proteus mirabilis were able to inhibit osteoclast formation and bone resorption, that could be key osteoporosis and rheumatoid arthritis characterized by excessive osteoclast activity and bone loss [126]. These OMVs achieved a reduction in bone loss in experimental osteoporosis and collagen-induced arthritis. Mechanistically, OMVs downregulated miR-96-5p, leading to increased Abca1 expression and mitochondria-dependent apoptosis [126].

In diagnostics, bEVs offer promising biomarkers for disease surveillance and precision medicine. Their molecular cargo reflects the physiological or pathological state of their parent microorganisms, which are often dysregulated during disease. Disease-specific bEV signatures—comprising unique miRNAs, proteins, or lipids—have been identified in biofluids like serum, urine, saliva, and feces, making them suitable for non-invasive or minimally invasive diagnostics. These diagnostic applications are particularly compelling in chronic inflammatory diseases, cancer, and infections where early and specific detection remains challenging [115]. For example, based on metagenomic analysis, five types of bacteria phyla Firmicutes, Actinobacteria, Proteobacteria, phyla Bacteroidetes, and Verrucomicrobia detected in urine bEVs show great potential as biomarkers for the diagnosis of colorectal cancer, or in blood proteomics analysis of Firmicutes and Bacteroidetes in bEVs of the host intestinal bEVs are found to be reduced in patients with psychosis compared to healthy, and there are many other examples [104,127,128].

Despite these advances, challenges remain—particularly regarding the standardization of bEV isolation, purification, and characterization. There is currently no consensus on the optimal techniques for isolating bEVs from clinical or environmental samples, with methods ranging from ultracentrifugation and size exclusion chromatography to microfluidic platforms. Moreover, batch variability, scalability, and regulatory issues further complicate their translation into clinical applications.

Microbial EVs stand at the intersection of microbial pathogenesis, immune modulation, and therapeutic innovation. Their inherent capacity to carry and deliver a diverse array of bioactive molecules positions them as both culprits and curatives in the context of inflammation. As the field advances, the multifaceted nature of bEVs is becoming increasingly evident. They not only function as natural delivery systems for microbial antigens and virulence factors but also hold promise as engineered nanocarriers for therapeutic agents, including vaccines, antimicrobial peptides, and nucleic acid-based therapies. Their presence in biofluids and their dynamic composition in response to environmental changes underscore their diagnostic potential as non-invasive biomarkers. EVs offer a unique lens through which to explore the dynamic interplay between microbes and hosts. Their dualistic nature—simultaneously pathogenic and protective—underscores their importance in shaping inflammatory responses and highlights their value as next-generation tools in precision medicine. Unlocking the therapeutic and diagnostic promise of microbial EVs will not only deepen our understanding of microbial biology but also open new avenues in the treatment and monitoring of human disease.

## 8. Roles in Immune Response

In multicellular organisms, maintaining immune homeostasis and responding to external threats relies on an intricate network of communication between cells. Classically, this intercellular crosstalk was understood to occur through direct cell–cell contact or via the secretion of soluble factors such as cytokines, chemokines, and hormones. However, in the past two decades, a novel mode of communication has gained prominence: EVs, particularly exosomes. These vesicles, once dismissed as cellular debris, are now recognized as potent biological messengers with key roles in both physiological and pathological immune (but not only) contexts [129]. Exosomes are a subclass of EVs ranging from 30 to 150 nm in diameter, originating from the endosomal compartment. They are released into the extracellular environment upon the fusion of multivesicular bodies (MVBs) with the plasma membrane. Virtually all cell types, including immune cells, release exosomes as part of their normal and stress-related activities. These vesicles carry a diverse cargo of proteins, lipids, and nucleic acids, reflecting the state and origin of the parent cell. Because of this, exosomes are increasingly viewed not only as biomarkers for disease but also as potential vehicles for therapeutic delivery.

In the context of immunology, exosomes exhibit a dual role. Indeed, the innate immune system forms the first line of defense and comprises a network of cells, including monocytes, macrophages, dendritic cells, neutrophils, and natural killer cells, facilitating the earliest interactions between the host and pathogens. Upon entry of a foreign object, recognition of the entry of an invader, cell–cell communication is critical for swiftly spreading the message of infection and enabling the innate immune system to mount a broad response against the pathogen. Until recently, cytokines and chemokines have been extensively studied for their role as messengers in innate immunity. However, recent research has revealed that exosomes are also vital in this communication [130]. On one hand, they can enhance immune responses by presenting antigens, activating immune cells, and propagating inflammatory signals. On the other hand, they can promote immune tolerance or suppression by delivering inhibitory molecules or immunosuppressive cytokines, such as TGF-β and IL-10. This versatility has sparked interest in exploiting exosomes for immune modulation, cancer immunotherapy, and the treatment of autoimmune disorders [131,132]. Additionally, exosomes have emerged as crucial players in the immune response to infections. They can transfer viral or bacterial components between cells, modulate the activity of pattern recognition receptors, and either enhance or mitigate inflammation depending on their origin. In light of these findings, the study of exosomes has rapidly evolved from descriptive studies to translational research with clinical potential [105].

Exosomes play a multifaceted role in orchestrating both innate and adaptive immune responses by acting as vehicles for intercellular communication. Their immunomodulatory potential arises from their ability to carry and deliver immunologically relevant molecules, including antigens, cytokines, costimulatory molecules, lipids, and various classes of RNAs. Their effects depend largely on the cellular origin, physiological context, and specific cargo composition.

### 8.1. Roles in Innate Immunity

The innate immune system, as the first line of defense, relies on rapid, non-specific mechanisms to detect and neutralize pathogens. Exosomes significantly contribute to innate immune regulation by facilitating communication between innate immune cells such as macrophages, neutrophils, dendritic cells (DCs), and natural killer (NK) cells.

Macrophage-derived exosomes are well-studied for their pro-inflammatory capacity. Exosomes released from lipopolysaccharide (LPS)-stimulated macrophages are enriched in IL-1β, TNF-α, and other cytokines, as well as microRNAs like miR-155, which modulate TLR and NF-κB signaling in recipient immune cells [133]. These exosomes enhance the activation of the NLRP3 inflammasome and promote the recruitment of immune cells to the site of infection. Macrophage-derived exosomes also carry antimicrobial peptides and enzymes that directly degrade bacterial components [134]. For neutrophil-derived exosomes, they play a critical role in inflammation and pathogen elimination. These vesicles contain myeloperoxidase (MPO), neutrophil elastase, cathepsin G, and lactoferrin, which are involved in microbial killing [135]. Furthermore, neutrophil-derived exosomes modulate endothelial cell function, contributing to vascular leakage and leukocyte transmigration during inflammation [136]. Importantly, neutrophil-derived exosomes can also influence adaptive immune cells by modulating dendritic cell maturation and cytokine production [106]. Regarding the Dendritic cells (DCs), professional antigen-presenting cells, also release exosomes (DEXs) that play pivotal roles in both innate and adaptive immunity. Immature DC-derived exosomes primarily contribute to immune surveillance by presenting pathogen-associated molecular patterns (PAMPs) via Toll-like receptors (TLRs), as well as HSP70 and HSP90 proteins that stimulate NK cell activity. In contrast, mature DCs release exosomes enriched in MHC-I/II, ICAM-1, and co-stimulatory molecules like CD80 and CD86, which are crucial for T cell priming [106]. Natural killer (NK) cell-derived exosomes possess cytolytic activity. These exosomes carry perforin, granzyme A/B, and FasL, which can induce apoptosis in tumor or virally infected cells. In addition to their cytotoxic properties, NK-cell-derived exosomes modulate the activity of other immune cells [137]. For example, NK-cell-derived exosomes can increase the antigen-presenting capacity of DCs or promote the polarization of macrophages toward a pro-inflammatory phenotype (M1) [111].

These findings on the innate immune system and exosomes highlight that exosomes are not merely byproducts of innate immune activation but active mediators that reinforce, amplify, or sometimes resolve inflammatory responses, depending on the context and stimulus. They can act locally at the site of infection or distantly via circulation, thereby serving as systemic immunological messengers.

### 8.2. Roles in Adaptive Immunity

Exosomes also exert significant influence over the adaptive arm of the immune response, which is characterized by antigen specificity and immunological memory. B cells and T cells engage in extensive exosomal communication to coordinate with adaptive immunity, via the dendritic cells.

B cell-derived exosomes (B-Exos) express MHC class II, CD19, CD81, and tetraspanins, and are known to present antigens to T helper (Th) cells. These vesicles can also transfer antigens and costimulatory molecules to follicular dendritic cells (FDCs), contributing to the germinal center reaction and antibody affinity maturation. Furthermore, B-Exos can modulate immune tolerance by transferring regulatory miRNAs, such as miR-150 and miR-155, to other immune cells [138,139].

T cell-derived exosomes (T-Exos) also serve dual functions. Exosomes from activated CD4+ T cells have been shown to carry cytokines (IL-2, IFN-γ for example) and transcription factors that influence antigen-presenting cells (APCs) and bystander T cells. CD8+ T cell-derived exosomes can contain perforin, granzyme B, and FasL, enabling them to exert cytotoxic effects on target cells independently of direct cell–cell contact [140,141]. Moreover, T-Exos carry miRNAs and surface molecules that modulate dendritic cell maturation and MHC expression. A particularly important subset of T cells, regulatory T cells (Tregs), utilize exosomes to mediate immunosuppression. Treg-derived exosomes often express CTLA-4, CD25, CD39, PD-L1, and contain IL-10 and TGF-β [142]. These components act on dendritic cells to downregulate costimulatory molecules or on effector T cells to inhibit proliferation and cytokine production [117]. Furthermore, Treg-derived exosomes contain miRNAs (e.g., miR-142-3p, miR-150) that suppress pro-inflammatory gene expression in recipient cells [138].

Exosomes also play a central role in antigen presentation and memory generation. DEXs facilitate cross-presentation of antigens to CD8+ T cells via MHC-I complexes and indirectly promote CD4+ T cell activation through the transfer of MHC-II/peptide complexes to naïve DCs [143]. This process enables a single antigen-loaded DC to trigger a broader immune response [106].

Exosomes have been implicated in both the resolution and exacerbation of adaptive immune responses. While some exosomes enhance T and B cell activation, others may suppress the immune activity, and sometimes even immune surveillance in the context of tumor. Tumor-derived exosomes expressing PD-L1 or FasL can inhibit CD8+ cytotoxic responses and induce T cell apoptosis [144].

Together, these findings underscore the crucial role of exosomes in shaping the adaptive immune response. The adaptive immune modulation by exosomes is a dynamic and tightly regulated process that balances immune activation and tolerance. As active participants in immune signaling, exosomes offer a unique opportunity for therapeutic targeting and modulation of immune memory, tolerance, and activation.

### 8.3. Exosomes and Sepsis

Sepsis is a life-threatening condition that arises when the body’s response to infection becomes dysregulated, leading to systemic inflammation and subsequent organ dysfunction. It represents a major global health challenge, contributing significantly to morbidity, mortality, and healthcare costs. In the United States alone, sepsis accounted for over $20 billion in hospital expenses and continues to be one of the leading causes of death among hospitalized patients. The incidence of sepsis is rising, likely due to aging populations, increased awareness and detection, and improved documentation practices [145].

Historically, sepsis was defined through the lens of systemic inflammatory response syndrome (SIRS), which emphasized the presence of infection-induced inflammation. However, this framework lacked specificity and failed to capture the complexity of the host–pathogen interaction. In 2016, the Third International Consensus Definitions for Sepsis and Septic Shock (Sepsis-3) redefined sepsis as “life-threatening organ dysfunction caused by a dysregulated host response to infection” [122]. This pivotal update shifted the focus from inflammation alone to a more integrated perspective incorporating cellular, biochemical, and immunologic dysregulation. Importantly, survivors of sepsis often face long-term impairments including cognitive deficits, physical disability, and psychological distress [122]. Given the pivotal role exosomes play in shaping immune responses, their involvement in the progression, or resolution of sepsis is gaining attention.

#### 8.3.1. Exosomes as Diagnostic Tools in Sepsis

Sepsis remains a major challenge in modern medicine due to its heterogeneity, rapid progression, and the lack of specific and early diagnostic biomarkers. Conventional biomarkers such as C-reactive protein (CRP), procalcitonin (PCT), and interleukin-6 (IL-6) provide some information about systemic inflammation but often lack sensitivity and specificity for early-stage or organ-specific sepsis detection. In recent years, exosomes have emerged as promising biomarkers in this context. During sepsis, both the quantity and content of circulating exosomes are significantly altered. For instance, levels of exosomal CD63 (a tetraspanin commonly used as an exosome marker) are significantly elevated in septic patients and positively correlate with organ dysfunction and mortality, as measured by SOFA scores (organ failure measurement) [146].

More specifically, exosomal microRNAs (miRNAs) have been proposed as non-invasive and dynamic biomarkers. miR-146a, miR-155, and miR-223 are among those shown to be dysregulated in sepsis and are involved in modulating Toll-like receptor (TLR) signaling and cytokine production [147]. These miRNAs can reflect the inflammatory status of the host and offer prognostic value beyond classical biomarkers. Additionally, some studies demonstrate that exosomal RNA and protein cargo vary according to the phase of the immune response—pro-inflammatory versus immunosuppressive—thus enabling not only diagnosis but also the monitoring of disease progression and response to treatment [148]. For instance, exosomes derived from endothelial or immune cells carry damage-associated molecular patterns and cytokines that can be detected in peripheral blood early in the course of infection [104].

Advanced exosome detection technologies, such as nanoparticle tracking analysis, flow cytometry, and high-throughput RNA sequencing, have facilitated their integration into clinical research [149]. However, challenges remain regarding the standardization of isolation and quantification methods, which are essential for reproducible diagnostics [125]. Nevertheless, the use of exosomes as biomarkers has the potential to revolutionize sepsis diagnosis by allowing for rapid, accurate, and organ-specific assessment, thereby enabling earlier and more targeted therapeutic interventions [125].

#### 8.3.2. Therapeutic Potential of Exosomes in Sepsis

In addition to their diagnostic utility, exosomes hold significant promise as therapeutic agents and delivery vehicles in sepsis. As seen previously, exosomes are capable of modulating immune responses and influencing cell survival in recipient cells through the transfer of bioactive molecules. These properties have prompted intense interest in using exosomes, particularly those derived from mesenchymal stem cells (MSCs), as therapeutic tools in the management of sepsis and its complications. MSC-derived exosomes are among the most studied for therapeutic applications due to their potent immunomodulatory properties [150]. These exosomes contain anti-inflammatory cytokines, regulatory miRNAs, and proteins that can attenuate systemic inflammation, reduce oxidative stress, and inhibit apoptosis in multiple organs. In preclinical models of sepsis-induced cardiomyopathy, MSC-derived exosomes have been shown to improve cardiac function by downregulating pro-inflammatory signaling pathways such as NF-κB and by reducing the expression of TNF-α and IL-1β [151]. These exosomes also help preserve mitochondrial integrity and function, thereby protecting cardiomyocytes from septic injury [127].

Beyond cardioprotection, exosomes have demonstrated protective effects in sepsis-induced acute kidney injury and acute lung injury. Exosomes from MSCs or endothelial progenitor cells have been reported to attenuate tubular epithelial apoptosis, reduce neutrophil infiltration, and promote tissue regeneration in the kidneys and lungs [147]. Exosomes exert these effects by delivering miRNAs (miR-21, miR-146a) that target pro-inflammatory genes and modulate macrophage polarization toward an anti-inflammatory (M2) phenotype. Moreover, exosomes have the capacity to cross biological barriers and deliver therapeutic cargo directly to target tissues, enhancing their potential as drug delivery systems in systemic inflammatory conditions such as sepsis [149].

Another promising application is the use of engineered or loaded exosomes as targeted delivery systems. Exosomes can be artificially loaded with anti-inflammatory drugs, siRNAs, or CRISPR components to target specific molecular pathways involved in sepsis pathogenesis. For instance, exosomes loaded with miR-126 or miR-223 mimics have been used to reduce vascular permeability and modulate immune responses in septic animals [152]. This precision-targeted therapy could mitigate the detrimental systemic effects of broad-spectrum anti-inflammatory drugs while enhancing efficacy and safety.

Despite their therapeutic promise, several challenges remain. These include issues with large-scale exosome production, standardization of loading techniques, quality control, and regulatory approval. Nevertheless, the ability of exosomes to modulate immune responses, deliver therapeutic payloads, and target specific organs or cells makes them a highly attractive platform for next-generation therapies in sepsis. Ongoing clinical trials and translational research will be crucial in determining the real-world feasibility of exosome-based therapies [153].

In summary, exosomes serve as both promising biomarkers and innovative therapeutic agents in the management of sepsis. Their unique properties on the immune system regulation provide opportunities for improving early diagnosis, monitoring disease progression, and implementing precision medicine strategies that target the complex immunopathology of sepsis.

## 9. Roles in Disease Pathogenesis

### 9.1. Material Transport & Information Exchange

Exosomes play a critical role in material transport and intercellular communication by serving as natural carriers of bioactive molecules, including proteins, nucleic acids, lipids, and cytokines. Through the delivery of these molecular cargos, exosomes regulate cellular metabolism, modulate gene expression, and thereby influence both physiological and pathological processes [154]. Depending on the molecular context, this exchange can either promote tissue repair and homeostasis or contribute to the progression of disease.

Beyond molecular transport, exosomes are central mediators of intercellular signaling, affecting key biological pathways such as angiogenesis, apoptosis, inflammation, and antigen presentation [155]. By orchestrating these processes, exosomes function as pivotal regulators in maintaining cellular balance, but also as drivers of pathological remodeling in conditions such as cancer, cardiovascular disease, and chronic inflammation.

### 9.2. Disease Initiation and Progression

Exosomes have emerged as critical mediators in the initiation and progression of a wide spectrum of diseases due to their ability to transport bioactive molecules and modulate intercellular signaling. They contribute to the pathogenesis of autoimmune diseases such as rheumatoid arthritis (RA), systemic lupus erythematosus (SLE), type 1 diabetes mellitus (T1DM), and inflammatory bowel disease (IBD), where they facilitate immune dysregulation and chronic inflammation [156]. For example, recent spectroscopic analyses of plasma-derived EVs from T1DM patients revealed disease-specific biochemical alterations that were associated with the presence of diabetic sensorimotor peripheral neuropathy, highlighting the utility of EVs as sensitive indicators of disease progression beyond conventional blood components [15].

In cancer, exosomes can create a tumor-supportive microenvironment by promoting angiogenesis, immune evasion, and metastasis, while in chronic lung diseases, including asthma, chronic obstructive pulmonary disease (COPD), sarcoidosis, and tuberculosis, contribute to sustained inflammation, tissue remodeling, and impaired host defense [157].

A particularly significant role of exosomes is seen in neurodegenerative disorders such as Alzheimer’s disease, Parkinson’s disease, amyotrophic lateral sclerosis (ALS), and prion diseases, where they act as vehicles for the propagation of pathogenic proteins, including amyloid-β (Aβ) and tau, thereby exacerbating neuronal injury and disease spread [158]. Interestingly, exosomes can also exert neuroprotective functions under certain conditions by transferring neurotrophic factors, reducing oxidative stress, and supporting synaptic plasticity, underscoring their dual and context-dependent role in neurological disease.

Collectively, these findings highlight exosomes as both drivers of disease progression and potential targets for novel diagnostic and therapeutic strategies across diverse pathological states

### 9.3. Disease Transmission

Exosomes also play a pivotal role in disease transmission by acting as carriers of pathogenic information from infected or diseased cells to neighboring or distant healthy cells. These EVs can encapsulate and transport viral particles, bacterial components, or aberrant host-derived molecules such as misfolded proteins and oncogenic nucleic acids, thereby facilitating the spread of infection or pathological signaling beyond the site of origin [159]. In the context of infectious diseases, exosomes derived from pathogen-infected cells have been shown to modulate host immune responses, either by enhancing pathogen persistence through immune evasion or by exacerbating inflammation, ultimately worsening disease prognosis. Similarly, in cancer and other chronic conditions, diseased-cell-derived exosomes can disseminate malignant or dysregulated signals that reprogram healthy recipient cells, contributing to systemic progression and intercellular communication of pathology. Through these mechanisms, exosomes function not only as biomarkers of disease burden but also as active agents in the horizontal transfer of pathogenic material, underscoring their importance in understanding disease propagation and developing targeted therapeutic interventions.

### 9.4. Diagnostic and Prognostic Biomarkers

Exosomes are increasingly recognized as promising diagnostic and prognostic biomarkers due to their stability, accessibility in biofluids, and ability to mirror the physiological or pathological state of their parent cells. Their cargos—including RNAs (mRNA, miRNA, lncRNA, circRNA), proteins, lipids, and enzymes—undergo disease-specific alterations that can be detected in plasma, urine, saliva, and cerebrospinal fluid, providing minimally invasive opportunities for monitoring disease progression [160].

Advances in exosome isolation and characterization techniques, such as ultracentrifugation, size-exclusion chromatography, immunoaffinity capture, and emerging microfluidic platforms, have enhanced the sensitivity and specificity of exosome-based assays. In oncology, liquid biopsy approaches leveraging exosomal nucleic acids and proteins have demonstrated potential for early detection of tumorigenesis, assessment of metastatic risk, and prediction of therapeutic resistance. Similarly, in viral infections including HIV/AIDS, profiling of exosomal components has shed light on viral replication, immune modulation, and disease prognosis.

The development of omics technologies—such as exosomal transcriptomics, proteomics, and lipidomics—further strengthens their clinical utility by enabling comprehensive molecular profiling at the single-vesicle level. Collectively, these advances not only position exosomes as highly informative biomarkers but also underscore their potential to transform precision diagnostics and patient-tailored disease management.

### 9.5. Therapeutic Potential

Exosomes are emerging as highly versatile therapeutic agents, with growing interest in their use as natural drug delivery vehicles owing to their nanoscale size, intrinsic stability, biocompatibility, and ability to traverse biological barriers without eliciting significant immune rejection [161]. Their lipid bilayer structure protects therapeutic cargos, such as small molecules, nucleic acids, or proteins, from enzymatic degradation, thereby enhancing delivery efficiency and bioavailability.

In addition to their carrier function, exosomes exhibit inherent therapeutic potential by modulating immune responses, reducing inflammation, and promoting tissue regeneration [162]. Preclinical studies have demonstrated their application in diverse disease contexts, including cancer, where engineered exosomes can deliver chemotherapeutics or RNA-based therapeutics directly to tumor cells, as well as in neurodegenerative and cardiovascular diseases, where they facilitate the targeted transfer of neuroprotective or cardioprotective factors.

Furthermore, advances in bioengineering have enabled the modification of exosomal surfaces to enhance tissue specificity and therapeutic efficacy, positioning them as a next-generation platform for precision medicine. Collectively, these features underscore the therapeutic versatility of exosomes and highlight their potential to transform conventional treatment paradigms across a wide range of diseases.

### 9.6. EVs and Cancer Biology

Cancer progression is driven by a variety of pathological processes that manipulate surrounding tissues and cells. Often referred to as the ‘hallmarks of cancer,’ these processes include sustained proliferation capacities, angiogenesis, invasion and metastasis, and immune system evasion [163]. EVs are paramount mediators of communication between tumor cells and their environment and contribute greatly to the ability of cancers to fulfil these hallmarks and progress into devastating diseases [164]. Understanding the intricate roles that EVs play in these functions can potentially lead to advancements in tumor detection, staging, and treatment.

Mutations of proto-oncogenes and tumor suppressor genes permit unregulated and persistent proliferation of cancer cells and are fundamental to malignant transformation. Beyond intrinsic mutations, evidence has shown that tumor cells can horizontally transfer oncogenic material to normal recipient cells, propagating malignant traits [165]. For example, EVs secreted by human osteosarcoma cells were shown to be effectively assimilated by healthy murine fibroblasts in vitro, inducing a tumor-like phenotype with increased proliferative capacity, resistance to starvation conditions, and a propensity to migrate [166]. To explain these effects, Urciuoli et al. demonstrated the presence of tumorigenic human mRNAs (TNF-α, Interleukin-6 (IL-6), and Transforming Growth Factor-β (TGF-β)) in the murine fibroblasts after one treatment with osteosarcoma-EVs. Similarly, in Ewing sarcoma, the histone methyltransferase EZH2 mRNA was identified in tumor EVs and has been shown to induce a more aggressive malignant phenotype when present [167,168]. In Ewing sarcoma, the EWS-FLI1 fusion oncoprotein directly binds to the EZH2 promoter and upregulates its expression, contributing to a stem-like phenotype which supports tumor growth and metastasis. When Ewing sarcoma EVs were co-cultured with mesenchymal stem/stromal cells, they induced an increased expression of EZH2 [168]. However, when Ewing sarcoma EVs were co-cultured with osteoblasts, there was no effect on EZH2 level, and EZH2 was even decreased when co-cultured with osteoclasts. This effect may be explained by the fact that mesenchymal stem/stromal cells are the precursor cells to Ewing sarcoma and are thus more vulnerable to malignant transformation. It also may highlight possible selectivity in horizontal transfer. Another in vitro study using human breast cancer and glioma cell lines demonstrated the transfer of tissue transglutaminase (tTG) and fibronectin to healthy fibroblasts via EVs induced tumorigenic conversion [169]. They found that tTG and fibronectin are both required to activate mitogenic properties in recipient cells, and when they are inhibited, these effects are not observed. Horizontal transfer via EVs has also been shown in an in vivo mouse model using colorectal cancer EVs. In a study by Abdouh et al., intravenous injections of colorectal cancer EVs into mice resulted in neoplastic formations and lung metastases [170]. Histological analysis of these tumors demonstrated a colorectal cancer phenotype with expression of multiple characteristic markers (CK20, CEA, CDX2, AE1/AE3). These findings provide evidence that tumor EVs can transfer functional genetic material to non-malignant cells, reshaping the tumor microenvironment and promoting disease progression.

As these processes promote tumor growth, metabolic demand increases, necessitating the formation of new blood vessels to supply oxygen and nutrients. EVs play a central role in this process of angiogenesis, another major hallmark of cancer. The cargos and functions of tumor-derived EVs depend on the present tumor microenvironment. Under hypoxic conditions, tumor cells increase EV production and alter EV cargo to enhance angiogenic potential [9]. For example, hypoxia-induced EVs are enriched in pro-angiogenic factors such as VEGF, MMPs, ALS2, lncRNA MALAT1, and miR-135b [171,172,173,174,175]. Silva et al. cultured human lung adenocarcinoma cells in normoxic and hypoxic conditions, demonstrating that EVs produced by cells under hypoxic conditions caused increased endothelial cell migration and tube formation in vitro [171]. Further investigation revealed that transfer of the Rab5 guanine nucleotide exchange factor (GEF), ALS2, mediates the observed effects via activation of Rab5 and subsequent β-catenin signaling. A similar study of osteosarcoma-derived EVs demonstrated that EVs derived from cells cultured in an acidic pH (6.5) compared to neutral pH (7.4) led to increases in both length and branching of vessels in an in vivo chick chorioallantoic membrane (CAM) assay [175]. It is apparent that the functions and quantities of tumor-derived EVs are dynamic and contribute to major adaptations like angiogenesis.

Tumor vascularization not only supports continued survival and growth but also creates avenues for escape into distant sites. To directly access these vessels, tumors must first breach the surrounding extracellular matrix (ECM). EV cargos, including mixed metalloproteinases (MMPs) which degrade ECM structure, are vital mediators of this process [176]. Notably, membrane-type 1 MMP (MT1-MMP) is a transmembrane proteinase found in EVs secreted by multiple cancer types that can degrade collagen, fibronectin, laminin, and gelatin [177]. EVs isolated from human melanoma and fibrosarcoma cell lines contain MT1-MMP, and when these EVs were mixed with collagen or gelatin, they had degradative effects [178]. Additionally, this study demonstrated that MT1-MMP-containing EVs could activate pro-MMP-2, which further contributes to ECM remodeling effects. MT1-MMP-containing EVs can also be tethered to cancer cells via a protein called tetherin, which further enhances their ECM degradative effects and promotes cancer cell invasion [179]. Furthermore, MT1-MMP works synergistically as an ECM remodeling agent and pro-angiogenic factor, as it increases VEGF-A production in tumor cells, enhancing vascularization [180]. Another molecule called extracellular matrix metalloproteinase inducer (EMMPRIN) is found on the membranes of EVs from a wide variety of cancers, including breast cancer, pancreatic cancer, and leukemia [20]. EMMPRIN interacts with recipient cells, most often monocytic cells, and stimulates production of MMP-9, IL-6, and TGF-β1, all of which are potent drivers of tumor invasion and microenvironment alterations [181]. These studies highlight some key drivers ECM remodeling that permits local invasion of tumor cells, which is a critical step in the progression toward metastasis.

Once direct access to newly formed vessels is attained, EVs released by tumor cells can enter and circulate systemically, where their surface molecules, such as integrins and adhesion proteins, direct them to specific organs. Upon arrival, EVs reprogram the local microenvironment to favor tumor colonization and progression, serving as predecessors of metastasis. Hoshino et al. discovered that tumor-derived EVs determine organotropic metastasis, with specific integrins for unique organs, including ITGαvβ5, which binds Kupffer cells mediating liver tropism, whereas ITGα6β4 and ITGα6β1 bind lung-resident fibroblasts and epithelial cells, facilitating lung tropism [37]. In a mouse model of pancreatic ductal adenocarcinoma (PDAC), retro-orbital injection of PDAC-EVs every other day for three weeks followed by intra-splenic injection of PDAC cells led to increased liver metastasis 21 days following intra-splenic injection of tumor cells compared to non-EV-injected mice [182]. Additionally, this study showed that macrophage migration inhibition factor (MIF) found in the PDAC EVs prepares the liver as a site for metastasis by inducing fibronectin production by Kupffer cells through TGFβ, and by recruiting bone marrow-derived cells to the liver that establish a tumor-supportive microenvironment. Similar studies on osteosarcoma-derived EVs demonstrate that highly metastatic cell lines secrete three-fold more EVs than poorly metastatic lines, and that EVs produced by highly metastatic EVs can induce metastasis by chemotaxis and horizontal transmission of genetic material [183]. In these regards, EVs may serve as a window into the future of a tumor’s progression, providing clinical insight and opportunities for intervention.

**Figure 3:**
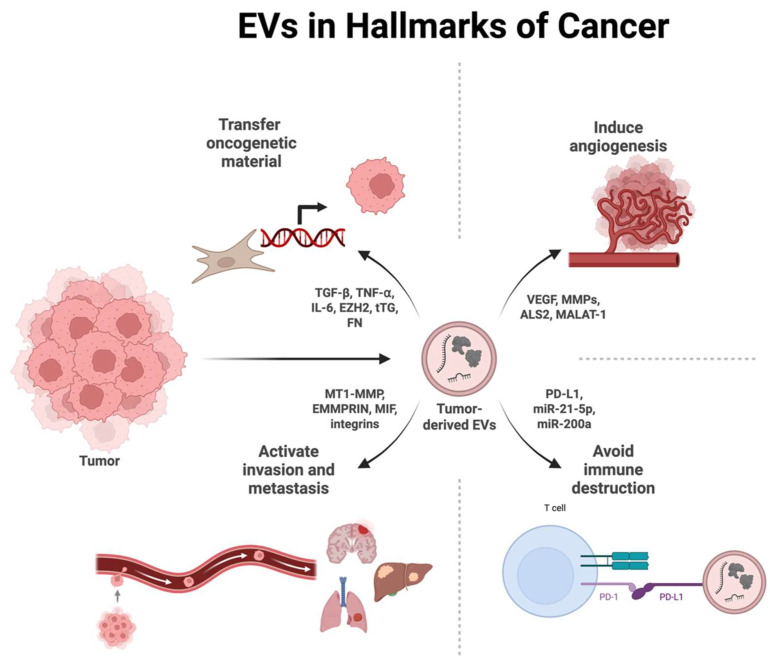
Schematic of the roles of EVs and their respective cargos in various hallmarks of cancer (oncogenic mutations, angiogenesis, invasion and metastasis, and immune system avoidance) (Created in BioRender. https://BioRender.com/0fbrvme, accessed on 15 December 2025).

In addition to promoting cell proliferation, inducing new vasculature, and mediating metastasis, EVs assist malignant tumor cells in evading the host immune response, allowing for unhindered cell propagation and proliferation. Malignant tumor cells can be detected and effectively eliminated by the immune system through the process of immunosurveillance. One way that tumor cells can subvert this immune response is through directly suppressing cytotoxic immune cells such as natural killer (NK) cells or CD8+ T-cells [184]. A well-characterized mechanism through which this occurs is through the interaction of programmed cell death-1 (PD-1) and programmed death ligand-1 (PD-L1), which are mainly expressed on immune cells and tumor cells, respectively [24]. However, evidence shows that tumor cells can leverage EVs to directly suppress cytotoxic T-cells via exosomal PD-L1 surface expression, deliver PD-L1 directly to target cells, or induce its expression in nearby tumor or stromal cells. Firstly, Chen et al. and Ricklefs et al., studying metastatic melanoma and glioblastoma, respectively, found that exosomal-surface PD-L1 can directly suppress the immune response by directly interacting with T-cell surface receptor PD-1 [185,186]. Secondly, Li et al. demonstrated that metastatic prostate cancer cells deliver PD-L1 to nearby tumor cells, amplifying the immune evasion response of tumor cells [187]. Interestingly, Li et al. found that the level of exosomal PD-L1 expression in metastatic prostate cancer correlates with a higher cancer grade. Similarly, Chen et al. showed that metastatic melanoma EVs express PD-L1 at higher rates than their primary cell line counterparts. These findings suggest some prognostic value of exosomal PD-L1 expression in predicting disease progression. Also, Yin et al. demonstrated that colorectal cancer cell-derived EVs can induce PD-L1 expression in nearby tumor or stromal cells [188]. This process was mediated by two key exosomal-derived miRNAs, miR-21-5p and miR-200a, and it resulted functionally in shifting macrophages into an anti-inflammatory M2 phenotype and suppressing the cytotoxic T-cell response. Herein, tumor-derived EVs functioned more indirectly by activating the PD-1/PD-L1 immune evasion mechanism in nearby cells. Whether it is through direct delivery of PD-L1 or induction of PD-L1 expression in nearby cells, tumor-derived EVs are large mediators of this immune evasion hallmark of cancer cells (Figure 3).

Given their central role in genetic reprogramming, angiogenesis, and metastasis, EVs have emerged not only as pathophysiologic drivers of cancer but also as promising candidates for clinical application. The same properties that make EVs effective agents of tumor progression—stability in biofluids, selective cargo loading, and targeted uptake—also render them attractive as biomarkers of prognosis and as vehicles for novel therapeutics. While current cancer liquid biopsies that detect circulating tumor cells or cell-free DNA are fairly insensitive, the detection of cancer-derived EVs may be the solution to these screening and monitoring tests. EVs are much more abundant in the serum than circulating tumor cells and are stable in biofluids as opposed to cell-free tumor DNA. Additionally, they contain a variety of cargos (DNA, RNA, proteins) that can be identified as markers for specific cancers [189,190]. A review by Andre et al. investigated potential EV-derived biomarkers for a variety of cancers, for example, EGFRvIII in glioblastoma-derived EVs, which can be used as both diagnostic and prognostic indicators, as many of these EV-cargos are functional and integral to the cancer’s pathophysiology [191]. Another clinical study found that serum from patients with glioblastoma contained a significantly increased concentration of EVs, compared to not only healthy controls but also patients with brain metastases and extra-axial brain tumors [192]. This study also observed a decrease in serum EV concentration following surgical resection, and a subsequent increase in patients with recurrent glioblastoma [192]. These examples in glioblastoma demonstrate two methodologies that can be used to screen or monitor cancers, using specific markers or total serum concentrations. Certain cancers are often caused by specific gene mutations, for example, the translocation of chromosomes 11 and 22 leading to EWS/Fli-1 fusion gene, which is present in over 95% of Ewing sarcoma cases. EWS/Fli-1 mRNA has been detected in Ewing sarcoma-derived EVs in multiple studies [193,194]. Thus, screening by way of EV cargo detection may be a readily available tool for similar cancers with characteristic mutations, or as monitoring of recurrence in previously genotyped tumors.

### 9.7. Roles in Tissue Injury and Repair

Tissue injury from trauma, degenerative disease, or surgical intervention activates inflammatory and reparative cascades. Cell-based therapies such as mesenchymal stromal cells (MSCs) offer regenerative promise but pose challenges in scalability, immunogenicity, and regulatory oversight. EVs, and particularly exosomes, represent a next-generation approach, harnessing the paracrine effects of MSCs in a cell-free, off-the-shelf format. Exosomes deliver bioactive cargo—including miRNAs, cytokines, lipids, and proteins—directly to injured tissues, modulating healing without the risks of cell engraftment. As interest in clinical applications grows, regulatory agencies such as the FDA and EMA are working to define frameworks to govern their use.

EVs contribute to tissue repair through immunomodulation, angiogenesis, matrix remodeling, and stimulation of resident progenitor cells. These biological mechanisms support orthopedic, dermal, cardiac, and neurologic repair contexts where restoration of tissue architecture and immune balance are critical.

### 9.8. Preclinical and Early Clinical Applications

Animal models consistently demonstrate the efficacy of EVs in cartilage, bone, tendon, dermal wound, and cardiac repair. In osteoarthritis models, MSC-derived exosomes reduce cartilage degeneration and support joint homeostasis. Early-phase clinical studies, though limited in size and geography, suggest safety and feasibility. In knee osteoarthritis specifically, case reports and pilot studies have shown symptomatic improvement, though the lack of randomized controlled trials prevents definitive conclusions. As of now, there is no peer-reviewed clinical evidence sufficient to support widespread clinical use of exosomes for musculoskeletal disorders in the U.S. or Europe.

### 9.9. Harmonization and Clinical Implications

The absence of harmonized standards contributes to regulatory ambiguity. Both FDA and EMA emphasize the need for potency assays, standardized nomenclature (ISEV guidelines), and validated reference materials. For clinicians, exosome therapies for musculoskeletal conditions, including knee osteoarthritis, remain investigational. Patients may only access them within formal clinical trials. Regulatory caution reflects unresolved safety concerns—such as immunogenicity, off-target effects, and reproducibility—while underscoring the potential of exosomes as off-the-shelf biologics.

Donor source (autologous, allogeneic, or perinatal) raises ethical challenges. Manufacturing requires advanced bioreactors, scalable purification technologies such as tangential flow filtration, and strict adherence to cGMP. Quantification methods (e.g., nanoparticle tracking analysis, flow cytometry) are widely used but not harmonized, limiting regulatory approval. Cost and scalability remain significant challenges.

EVs represent a paradigm shift in regenerative medicine, offering therapeutic potential without the risks associated with cell transplantation. Their ability to modulate immune responses, stimulate angiogenesis, and support tissue repair makes them particularly relevant in musculoskeletal medicine. However, regulatory frameworks by both the FDA and EMA currently restrict their use to investigational studies. A harmonized approach emphasizing standardization, potency metrics, and rigorous safety evaluation will be essential to translate exosome science into clinical reality.

### 9.10. MSC-EVs: Unlocking Clinical Potential and Overcoming Translational Challenges

MSC-EVs have profound potential to treat a variety of conditions via their tissue regeneration and immunomodulatory effects, but face many translational challenges due to their heterogeneous nature and limited regulatory guidelines as a new therapeutic modality. Despite their novelty, technological advances in EVs manufacturing and isolation have allowed for the development of a cGMP framework by improving standardization and increasing EVs scale. These include transitioning from the classical 2D cell culture system to more physiologically relevant 3D culture [195,196]. Of the available 3D culture options, hollow fiber reactors with an automated 3D closed system for EVs collection show the most efficiency by maximizing scale and efficacy and minimizing time and cost required [197,198,199]. Closed systems are critical in this process by ensuring sterility and EVs quality. Recently, the most commonly used cGMP-compliant bioreactor systems are hollow fiber, stirred tank, and microcarrier bioreactors, each with its own strengths and weaknesses affecting scale, workload, cost, and EVs quality [200].

In addition to increasing EVs scalability and physiological relevance with 3D bioreactor systems, strict quality control guidelines must be followed to ensure the final MSC-EVs product meets the FDA’s standards concerning sterility, purity, efficacy, identity, and reproducibility. These factors can be impacted by every step in the production process including the origin of MSCs, how they are seeded into the 3D culture system, culture media composition, confluency and duration of the culture when EVs are isolated, the method used to isolate the EVs, and the storage conditions leading to patient administration, and administration route (among many other variables). Although ultracentrifugation (UC) and tangential flow filtration (TFF) are the preferred cGMP-grade EVs isolation options, TFF is more time efficient and higher yield as the final EVs isolate is purer and can easily be concentrated in a cryoprotectant of choice in a closed system compared to UC or smaller scale methods such as size exclusion chromatography [200].

Although quality requirements have not been delineated by the FDA yet, MSC-EVs fall under the biologics category and thus must follow similar strict quality standards and validation [201]. This is especially challenging in MSC-EVs manufacturing due to the variety of options available in manufacturing, starting with MSCs sourcing. Donor tissue, demographics such as age, race, gender, various health conditions, can play a significant role in the composition and functionality of MSCs and resulting EVs produced. The tissue of origin can also affect these characteristics and must be considered based on the intended clinical indication of the MSC-EVs therapeutic product. As such, batch-to-batch inter-donor validations like donor screening and master bank generation must be performed, followed by validation steps after MSCs harvest, EVs manufacturing, EVs isolation, and final MSC-EVs therapy release. These validation steps include assays confirming the identity, purity, reproducibility, sterility, and potency of EVs, using approaches delineated in recent publications and MISEV guidelines [202,203,204,205,206].

In general, MSC-EVs must be proven safe, pure, and potent in addition to having detailed characterization to enter the clinic. Their safety profile also needs to demonstrate minimal risk for organ damage, infection, malignancy, fatality, or injection reactions [207]. EVs therapeutic testing in animal models historically shows minimal general toxicity, and safety profiles of nanoparticle transfer in patients via blood or plasma transfusions are also known to have minimal risk [208,209,210,211,212,213,214,215,216,217,218]. Thus, MSC-EVs are poised to perform very well in translation to the clinic regarding toxicity. Despite this, toxicity testing criteria have yet to be set by the FDA as more direct analyses need to be established to ensure any toxicity is due to EVs specifically. This includes adequate functional profiling, which is less straightforward because EVs are heterogenous mixtures of nanosized particles with varying properties and resulting mechanism of action.

As with other biologic therapies and regenerative medicine, immunogenicity and tumorigenicity toxicity assessments must also be performed on MSC-EVs prior to clinical administration. Remarkably, most preclinical assessments show minimal immunogenicity and occasional mild immune responses depending on the source of EVs and animal model tested, despite known MHC and other immunoreactive components on their surfaces [219,220,221,222,223]. MSC-EVs are also known to have significantly less risk of immunogenicity, oncogenicity, and infection compared to MSC therapy because MSC-EVs are not capable of forming ectopic tissue or inducing malignant proliferation or differentiation in host cells. It is still necessary to use novel tools for the evaluation of these risks, including potential tumorigenicity based on evidence from Vallabhaneni et al. showing that human MSC-EVs augmented MCF-7 tumor growth and angiogenesis in a mouse study [224]. This does not mean that all MSC-EVs pose this risk, as many variables such as EVs source, state of assembly, size, and contents can influence their tumorigenicity. Specifically, using the soft agar colony formation assay can help delineate this risk in vitro [225].

MSC-EVs, as with all EVs therapies, are at the beginning stages of entering the clinic and thus do not have established regulatory requirements. EVs have been classified under five major therapeutic categories that each require full premarket approval in the US: (1) naïve EVs from naïve sources, (2) naïve EVs from modified sources, (3) modified EVs from modified sources, (4) naïve EVs with added cargo, and (5) liposomes [207]. Even though biogenic EVs are poorly defined due to their natural heterogeneity, these therapeutic categories of EVs will likely be characterized by their broad population activity

The EVs source and therapeutic indication will also likely impact the regulatory guidelines followed as each step in the manufacturing process impacts their safety profile and therapeutic efficacy in vivo. EVs isolation methods described previously are not EVs-specific, and this is further complicated by the unclear definition of EVs that could indicate the use of exosomes, microvesicles, apoptotic bodies, or a mixture thereof in the final therapeutic product. This necessitates the development of more specific regulatory standards as they relate to purity, safety, and efficacy.

In spite of these hurdles, MSC-EVs have dominated the EVs-based clinical trials reported to clinicaltrials.gov since 2010, especially for the treatment of respiratory diseases [226]. Most commonly isolated from bone marrow or umbilical cord MSCs, this MSC-EVs clinical trial data will help pave the way for a clearly defined regulatory framework for MSC-EVs moving forward.

## 10. EVs and Photobiomodulation

EVs and photobiomodulation (PBM) are two innovative modalities in regenerative medicine with significant synergistic potential. EVs are nanoscale particles (30–150 nm) released by cells that facilitate intercellular communication by transporting proteins, lipids, and nucleic acids. They are key regulators of physiological and pathological processes, including tissue repair and immune modulation.

PBM is a non-invasive technique that uses specific wavelengths of light (primarily red and near-infrared) to stimulate cellular processes. The therapeutic convergence of EVs and PBM represents a novel approach: PBM can prime cells for enhanced functionality and increased EV production, while EVs act as sophisticated transporters for the paracrine factors stimulated by PBM. This combined strategy offers promising opportunities for treating complex diseases, from chronic wounds to degenerative conditions, by modifying fundamental biological reactions. A summary of the effects of photobiomodulation on Extracellular Vesicle Production and Characteristics are shown in Table 1.

### 10.1. Fundamental Mechanisms of Photobiomodulation (PBM)

PBM operates through photophysical and photochemical reactions initiated by light wavelengths between 400 and 1400 nm. It is a non-thermal process that engages multiple physiological mechanisms, beginning with the absorption of photons by mitochondrial chromophores like cytochrome c oxidase [227]. This triggers a cascade of effects [228,229,230,231,232,233,234,235]:

- Enhanced Mitochondrial Function: Increased ATP production and modulation of reactive oxygen species (ROS).
- Immunomodulation: Context-dependent modulation of the NF-κB pathway, reducing pro-inflammatory cytokines (TNF-α, IL-1β, IL-6) in inflammatory states or boosting immune responses in immunosuppressed conditions.
- Cellular Effects: Polarization of macrophages towards an anti-inflammatory M2 phenotype, balancing of T-cell subsets, and enhanced function of dendritic cells.
- Tissue Repair: Activation of signaling pathways (TGF-β, MAPK/ERK, PI3K/Akt) that influence cell proliferation, migration, and angiogenesis.

The effects of PBM are fundamentally dose-dependent and follow a biphasic response (hormesis). Lower doses stimulate desired effects, while higher doses can be ineffective or inhibitory [236]. Parameters such as wavelength, fluence (energy density), and power density must be carefully optimized. Longer wavelengths (800–1000 nm) penetrate tissues more deeply, enabling treatment of both superficial and deep structures.

### 10.2. Interactive Effects Between Photobiomodulation and EVs

The intersection of PBM and EV biology is a promising area of research. Studies show that PBM can significantly influence both the production and composition of EVs [237,238,239,240,241,242,243,244,245,246,247,248].

- Enhanced Production: PBM acts as a physical primer for cells. In human adipose-derived stem cells (hADSCs), PBM at an optimal dose (5 J/cm^2^, 830 nm) resulted in a 6.25-fold increase in EV concentration compared to controls. This demonstrates PBM’s potential to boost EV yield for therapeutic applications.
- Modified Cargo: While EV size often remains unchanged, PBM can alter their functional cargo. Studies report upregulation of beneficial factors like RANKL (in osteoblasts) and anti-apoptotic protein BCL-2, alongside downregulation of pro-apoptotic genes (BAX, Caspase-3).
- Synergistic Therapeutic Effects: The combination of PBM and EV therapy often exceeds the benefits of either alone. In models of spermatogenesis arrest and PCOS, the combination improved sperm parameters, oocyte maturation, and antioxidant status while reducing apoptosis and ROS. This synergy suggests EVs mediate and amplify the therapeutic effects of PBM.

**Table 1:** Effects of Photobiomodulation on Extracellular Vesicle Production and Characteristics.

Aspect	Effect of PBM	Cell Type/Model	PBM Parameters (Examples)	Mechanism/Remarks	Reference
**EV production & yield**	↑ Increase in EV concentration (up to 6.25 times)	Human adipose stem cells (hADSCs)	830 nm, 5 J/cm^2^	Biphasic dose effect: Optimal effect at 5 J/cm^2^, higher doses (10 J/cm^2^) less effective	[238]
	↑ Increase in the secretion of angiogenic factors	Mesenchymal stem cells (MSCs)	Various, including 810 nm	PBM as a “priming” stimulus for MSCs	[249]
**EV size & distribution**	↔ No significant change in size distribution	Human adipose stem cells (hADSCs)	830 nm, 2.5–10 J/cm^2^	Size and distribution of EVs remained stable despite increased yield	[238]
**Composition & cargo**	↑ Up-regulation of RANKL in EVs	MG-63 Osteoblast-like cells	808 nm Diode laser	Possible mechanism for improved bone regeneration	[250]
	↑ Increase in anti-apoptotic proteins (BCL-2)	Ovocytes in PCOS	Not specified	Combination therapy PBM + EVs from umbilical cord blood	[246]
	↓ Downregulation of pro-apoptotic genes (BAX, Caspase-3)	Ovocytes in PCOS	Not specified	Combination therapy PBM + EVs from umbilical cord blood	[246]
**Functional properties**	↑ Improved wound healing	MSCs (in vivo)	Various	Combination of PBM + MSC-EVs shows synergistic effects	[249]
	↑ Improved pulp regeneration	MSCs (in vivo)	Various	Combination of PBM + MSC-EVs shows synergistic effects	[249]
	↑ Increase in cell migration	Human gingival fibroblasts	808 nm Diode laser	PBM alone is more effective than combination with PDLSC exosomes	[251]
	↑ Increase in cell migration	Human gingival fibroblasts	808 nm Diode laser	PBM alone is more effective than combination with PDLSC exosomes	[251]
	↑ Improved oocyte maturation	Ovocytes in PCOS	640 nm, 0.032 W/cm^2^, 1.85 J/cm^2^	Combination of PBM + EVs from umbilical cord blood	[246]
**Anti-apoptosis & cell protection**	↑ Increased cell survival rate	MSCs (in vitro)	Various	PB PBM priming reduces apoptosis	[249]
	↓ Reduction in ROS (reactive oxygen species)	Ovocytes in PCOS	Not specified	Combination therapy PBM + EVs from umbilical cord blood	[246]

### 10.3. Challenges and Future Directions of PBM

Despite the promising potential, several challenges must be addressed for clinical translation:

- Standardization: Both modalities lack standardization. Optimal PBM parameters (wavelength, fluence) and EV protocols (source, isolation, dosage) must be defined for specific applications [252,253,254].
- Safety and Biphasic Response: The biphasic dose–response of PBM means improper dosing can be inhibitory. The dualistic role of EVs in both promoting and suppressing disease (e.g., in cancer) requires careful evaluation [232,253,255].
- Manufacturing and Regulation: Scalable production of clinical-grade EVs and standardization of PBM devices present significant hurdles for widespread clinical use.

Future research should focus on: mechanistic studies to understand how PBM modifies EV cargo; rigorous standardization of parameters; preclinical safety studies; and well-designed clinical trials to assess efficacy.

The combination of EVs and photobiomodulation represents a transformative frontier in regenerative medicine. The interactive effects—where PBM enhances production and modifies the composition of EVs, and EVs subsequently mediate and amplify the therapeutic effects of PBM—create a powerful synergy. While challenges in standardization and manufacturing remain, the evidence provides a strong foundation for continued development. Harnessing this combination has the potential to yield novel, effective, and minimally invasive treatment strategies that leverage the body’s innate regenerative capabilities for a wide spectrum of diseases.

## 11. Regulatory Considerations: FDA and EMA

The FDA regulates exosomes as biological drugs under the Center for Biologics Evaluation and Research (CBER). They require either an Investigational New Drug (IND) application for research or a Biologics License Application (BLA) for commercial approval. Currently, no exosome product is FDA-approved for any clinical indication. Exosomes do not qualify for the 361 HCT/P minimal manipulation exemption, meaning they cannot be marketed as human tissue products. Manufacturing must comply with cGMP, with standards for sterility, identity (CD9, CD63, CD81), purity, and potency assays. Enforcement actions have been taken against companies for non-compliance

In Europe, the EMA categorizes exosomes as Advanced Therapy Medicinal Products (ATMPs) under the oversight of the Committee for Advanced Therapies (CAT). This designation places them in the same category as gene therapies, somatic cell therapies, and tissue-engineered products, reflecting their complexity and associated regulatory challenges [256]. To qualify for clinical development, sponsors must provide comprehensive quality and manufacturing data, including characterization of exosomal identity markers such as CD9, CD63, and CD81, quantification using nanoparticle tracking analysis or equivalent technologies, and evidence of batch-to-batch reproducibility [47]. Furthermore, non-clinical pharmacodynamics and toxicology studies are required to demonstrate biological activity and rule out safety concerns, while stability data must support long-term storage, transport, and administration conditions [256].

The EMA also mandates a risk management plan (RMP) for all ATMPs, requiring developers to conduct extensive post-marketing pharmacovigilance and safety monitoring to detect delayed or unanticipated adverse effects [256]. While no exosome-based therapy has yet been approved in the European Union, the EMA has provided scientific advice to several developers through its PRIority MEdicines (PRIME) scheme. PRIME is analogous to the FDA’s RMAT designation and is designed to accelerate the development of medicines addressing unmet medical needs by offering early dialogue with regulators, protocol assistance, and the possibility of accelerated assessment [256,257].

Despite these supportive mechanisms, significant challenges remain. The lack of harmonized potency assays and consensus on critical quality attributes continues to hinder trial comparability and regulatory confidence [50]. Furthermore, uncertainties surrounding large-scale production, reproducibility, and long-term safety require resolution before exosome-based therapeutics can enter routine clinical use. Nevertheless, the EMA’s structured ATMP framework provides a pathway that balances innovation and patient safety, ensuring that once sufficient clinical evidence is generated, exosomes may be translated into approved therapies across Europe [50,256].

## 12. Conclusions

EVs have garnered significant attention in recent years due to their fundamental role in intercellular communication and their complex molecular cargo, which mirrors the physiological or pathological state of their cells of origin. While their clinical potential is increasingly recognized, much of the current momentum lies in the preclinical and biological investigation of EVs. Key areas of focus include the elucidation of EV biogenesis, cargo sorting mechanisms, and the molecular determinants of vesicle uptake and biodistribution. At the same time, the field continues to face considerable challenges in the standardization and optimization of EV isolation, purification, and characterization protocols, which remain essential for reproducibility and downstream functional studies. The development of robust in vitro and in vivo models is critical for advancing our understanding of EV biology and for exploring their role in disease pathogenesis. As research continues to refine methodological approaches and uncover new biological insights, EVs are poised to become indispensable tools in both fundamental science and translational research.

## Data Availability

No new data were created or analyzed in this study. Data sharing is not applicable to this article.
